# Individual-level prediction models of societal costs and health-related quality of life in pediatric cerebral palsy: a population-based study from Spain

**DOI:** 10.1186/s12962-026-00743-y

**Published:** 2026-04-11

**Authors:** Diana Marcela Nova-Díaz, Paloma Arana-Rivera, Eduardo Sánchez-Iriso, Sergio Aguilera-Albesa, Diego Rivera

**Affiliations:** 1 Department of Economics, Public University of Navarra, Pamplona, Spain; 2 Paediatric Neurology Research Group, Navarrabiomed, Pamplona, Spain; 3 University Hospital of Navarra, Pamplona, Spain; 4 Department of Health Sciences, Public University of Navarra, Pamplona, Spain; 5 Instituto de Investigación Sanitaria de Navarra (IdiSNA), Pamplona, Spain

**Keywords:** Cerebral palsy, Health-related quality of life, Societal costs, Caregiver burden, Prediction models, Individual-level data, Resource allocation, Economic evaluation

## Abstract

**Background:**

Individual-level economic models more effectively capture the heterogeneity of clinical severity, caregiving needs, and social context in pediatric cerebral palsy (CP). We developed and validated predictive models to estimate how clinical, sociodemographic, socioeconomic, and health-related quality-of-life (HRQoL) factors affect disaggregated categories of societal costs. We then examined how these factors and cost components influence HRQoL outcomes for children with CP and their caregivers in Spain.

**Methods:**

Using data from 148 children with CP and their caregivers in a Spanish population-based registry, we estimated annual costs from a societal perspective. Cost components included healthcare, informal care, public subsidies, and out-of-pocket expenses (adjusted to €2023). We applied generalized linear models (GLMs) to model costs: one-part GLMs with gamma distribution and log link for most categories, and two-part models for intensive rehabilitation therapies (logistic regression for the probability of any cost, followed by a GLM for the positive costs). Child and caregiver HRQoL were modelled using ordinary least squares (OLS) and Tobit regression, respectively. Predictive performance was assessed via mean absolute error (MAE), root mean squared error (RMSE), and mean error (ME).

**Results:**

Greater functional impairment in children was the primary driver of higher costs and lower HRQoL. Socioeconomic factors, including caregivers’ job loss due to caregiving, low household income, and lower social class, predicted higher out-of-pocket spending and worse caregiver HRQoL. Latin American family origin was associated with higher total illness costs but lower informal care costs. Use of non-standard therapies (reported by 64% of families) increased overall societal costs and was linked to marginally better child HRQoL.

**Conclusions:**

Individual-level, disaggregated models can inform value-based healthcare assessments by capturing the complexity of pediatric disability. Our findings underscore the importance of caregiver burden and informal care in determining costs and well-being. We provide a *web-based Shiny calculator to enable practical application* of these predictive models.

**Supplementary Information:**

The online version contains supplementary material available at 10.1186/s12962-026-00743-y.

## Introduction

Paediatric cerebral palsy (CP) is a leading cause of childhood disability worldwide [1], with lifelong implications for health, care needs, and family well-being. Beyond clinical outcomes [2, 3], CP imposes substantial costs and emotional strain on families, especially in contexts of limited support and high care demands [4, 5]. Traditional economic evaluations often overlook the complex, interdependent nature of cost and Health Related Quality of Life (HRQoL) outcomes within families affected by paediatric disability [6, 7].

In recent years, the concept of Family Quality of Life (FQoL) has gained prominence as a framework to understand how children’s health conditions affect not only the individual but also the functioning, well-being [4, 6], and resilience of the entire family unit. In the health economics literature, related impacts on caregivers’ health and well-being are often described as *“spillover effects”* on quality of life, referring to the consequences that a child’s health condition may have on the HRQoL of family members or informal caregivers [8, 9]. While the spillover concept is typically applied to health utility impacts in economic evaluations, the FQoL framework provides a broader perspective that captures the multidimensional well-being of the family system, including emotional, social, and economic domains. In families of children with CP, caregivers frequently face increased financial pressure, mental health challenges, and reduced life satisfaction [10]. These effects are shaped by clinical severity, access to services, and the social determinants of health [11]. However, few studies have quantitatively modelled the joint outcomes of child and caregiver quality of life and societal costs at the individual level or analysed them as interdependent elements of the caregiving dyad. Understanding these interactions is essential to guide equitable and person-centred health policy.

The economic burden associated with CP is complex and multifaceted, encompassing not only direct medical costs but also informal caregiving, public subsidies, productivity losses among caregivers, and reductions in HRQoL [6, 12, 13]. Nevertheless, most economic evaluations continue to rely on aggregated or population-averaged models, which overlook the pronounced heterogeneity in clinical severity, care needs, and socioeconomic context [14, 15]. Conventional approaches often fail to capture how combinations of functional status, age, comorbidities, and family circumstances shape individual trajectories of cost and QoL [16, 17].

Given the growing interest in assessing the efficiency and equity of healthcare systems, particularly in settings with limited resources [18, 19], the integration of detailed economic modelling into the evaluation of CP is especially relevant. Individual-level models offer the ability to understand not only the variability in economic and quality-of-life burden across children, but also the disparities in access to services and support [20, 21]. Evidence from such models can inform policy decisions aimed at reducing inequalities in disability care, *optimizing resource allocation*, and *maximizing social value* [18, 22]. In this regard, individual-level models provide an essential tool for public health policies that seek to incorporate both direct and indirect costs, as well as HRQoL impacts, into cost-effectiveness frameworks [6, 23].

In this context, methodologies that incorporate individual heterogeneity and account for clinical and socioeconomic determinants of health have gained increasing relevance. These models simulate outcomes for each individual based on child-specific covariates, allowing for complex interactions between variables and enhancing the clinical realism and policy applicability of economic evaluations [23, 24]. In clinical fields such as oncology and cardiovascular disease, individual-level models have enhanced the precision of cost-effectiveness analyses and supported health technology assessments [19, 23]. However, their application in paediatric disability, particularly in CP remains limited. To our knowledge, no prior study has jointly modelled disaggregated cost components and HRQoL outcomes in this population using real-world, patient-level data.

Traditional aggregated models often rely on mean values and assume homogeneity in patient characteristics, which can obscure critical variation in both costs and health outcomes [25]. This limitation is particularly problematic in paediatric disability, where individual trajectories are shaped by complex, nonlinear interactions between functional status, caregiving dynamics, and social context [14]. Individual-level models overcome these constraints by simulating outcomes at the person level, allowing the incorporation of interaction terms, skewed distributions, and zero-inflated costs [23, 26]. This enhances both the precision and external validity of economic evaluations, and enables policy-relevant estimates that better reflect real-world heterogeneity—a critical advantage in paediatric disability research, where clinical and social complexity frequently coexists [22, 27].

Moreover, little is known about whether and how specific cost components, such as out-of-pocket (OOP) payments or informal caregiving, may influence HRQoL among children with CP or their caregivers [14, 28]. Understanding these bidirectional relationships is essential for designing interventions that reduce preventable expenditures while improving well-being. Beyond methodological innovation, this approach aligns with growing calls for equity and person-centred care in paediatric disability services, where economic and psychosocial dimensions are closely intertwined [12, 13].

This study addresses these gaps by developing and validating individual-level prediction models of both healthcare and social costs, as well as HRQoL outcomes, in children with CP. Using detailed data from a population-based registry in Navarra Spain, *we aim to (1) quantify the influence of clinical*,* sociodemographic*,* and socioeconomic factors on disaggregated cost categories*,* and (2) examine how these same characteristics and specific cost components shape HRQoL for both children and their primary caregivers*,* conceptualized as a caregiving dyad*. This approach also allows the analysis of caregiver HRQoL impacts that are often conceptualized in the health economics literature as *spillover effects* arising from the child’s health condition. We hypothesise that greater functional impairment and socioeconomic vulnerability are associated with higher individual-level costs and lower HRQoL, in part due to increased caregiver burden and limited access to services. Our findings provide empirical tools to support value-based resource allocation and inform equity-focused interventions that improve family well-being while reducing avoidable costs.

## Methods

### Study design and data source

This study employed a cross-sectional, individual-level design to develop and validate multivariable prediction models addressing two interrelated objectives. The first objective was to estimate the impact of clinical, sociodemographic, socioeconomic, and HRQoL characteristics on disaggregated components of healthcare and societal costs in children with CP. The second objective was to assess how these same individual-level characteristics, along with specific cost components, influence HRQoL outcomes. This bidirectional modelling framework enables a more comprehensive understanding of the patient-level determinants of both economic burden and quality of life in pediatric CP.

Data were drawn from the Pediatric Cerebral Palsy Epidemiological Registry of Navarra (Spain), a population-based database covering all confirmed pediatric CP cases in the region. The analysis used an annual time frame and included both children with CP and their primary caregivers, capturing the dual impact of disability at the individual and household level. This integrated perspective is particularly relevant in predictive modelling contexts, where the interplay between patient complexity, care needs, and broader social determinants shapes both costs and outcomes.

### Study population and data

This study used data from the Pediatric Cerebral Palsy Epidemiological Registry of the University Hospital of Navarra (HUN), a population-based database that includes all confirmed cases of CP in children residing in Navarra, a region in northern Spain with an estimated pediatric population of 134,898 individuals aged 0–18 years. According to 2023 estimates from the *EPCINA project (Estudio de la Parálisis Cerebral Infantil en Navarra)*, the regional prevalence of CP is 1.49 per 1,000 children, corresponding to approximately 201 cases.

Between July 2023 and December 2024, a total of 201 children with cerebral palsy (CP) were identified through the University Hospital of Navarra (HUN) and affiliated regional hospitals. Eligibility criteria included a confirmed diagnosis of CP, age between 3 and 18 years, permanent residence in Navarra, and the availability of a primary caregiver capable of providing informed consent and complete information on healthcare resource use and relevant outcomes. Children were excluded if their diagnosis remained unconfirmed during clinical follow-up or if they were under 3 years of age (*N* = 49), given the diagnostic uncertainty associated with early neurodevelopment, in line with the criteria established by the Surveillance of Cerebral Palsy in Europe (SCPE) network [20]. Additionally, four children (*N* = 4) who died during the study period were retained in the final sample [29].

The *final analytical sample consisted of 148 children and adolescents* with confirmed CP *and their primary caregivers*, thus representing the entire validated pediatric CP cohort in the region during the study period. The inclusion of caregivers was essential due to their central role in care coordination, informal care provision, and decision-making processes factors that directly affect healthcare expenditures and HRQoL outcomes. Participant identification was initially based on hospital records and was further expanded through coordinated outreach efforts involving regional health and disability service providers, ensuring comprehensive case ascertainment and minimizing selection bias.

### Measures

#### Clinical variables

To comprehensively characterize the clinical status of children with CP, we collected data on standardized functional classification systems that reflect the multidimensional nature of impairment in this population. These included the Gross Motor Function Classification System (*GMFCS*), Manual Ability Classification System *(MACS*), Communication Function Classification System (*CFCS*), Eating and Drinking Ability Classification System (*EDACS*), Bimanual Fine Motor Function (*BFMF*), Viking Speech Scale (*VSS*), and the Visual Function Classification System (*VFCS*). Each system provides ordinal, clinically validated measures of functional performance across distinct domains, enabling multidimensional stratification of severity.

In addition to functional profiles, other key clinical descriptors were incorporated to capture the etiological and neurodevelopmental complexity of CP. These included *CP type* (spastic, dyskinetic, ataxic, or mixed), *etiology* (prenatal, perinatal, or postnatal origin), and brain lesion characteristics based on the Magnetic Resonance Imaging Classification System (*MRICS*). The overall severity of impairment was operationalized using the CP *Impairment Index*, a composite indicator summarizing functional deficits across the aforementioned domains.

This granular clinical characterization was essential for the development of individualized predictive models of cost and HRQoL [30]. By incorporating these indicators into our multivariable modelling framework, we aimed to reflect the heterogeneity in care needs, service utilization, and outcomes within the paediatric CP population, thus enhancing both the precision and policy relevance of our findings [23].

#### Sociodemographic and socioeconomic variables

Detailed sociodemographic and socioeconomic data were collected to characterize the broader contextual factors that may influence HRQoL and healthcare costs of children with CP. Sociodemographic variables included *age and sex* of both the *child and primary caregiver*, as well as *geographic origin and place of residence* (Urban vs. Rural). These variables provide important contextual information regarding *patterns of care and demographic disparities*. *Socioeconomic indicators* included *the caregiver’s marital status*,* educational level*, and *current occupational status*. *Annual household income* and *family social class* were also documented as *proxy* indicators of *material living conditions and potential barriers to caregiving*.

To further *capture the socioeconomic burden of caregiving*, we recorded whether the *primary caregiver had left paid employment due to the child’s disability*. We also included *educational access indicators* by documenting the type of school attended by the child (*mainstream vs. special education)*, as educational setting may influence care needs and developmental outcomes. These variables were selected for their established or hypothesized relevance in shaping service utilization patterns, informal care provision, and HRQoL trajectories in paediatric CP. Their inclusion allowed for a more accurate estimation of heterogeneity in economic and health outcomes in individual-level models.

#### Health-related quality of life and caregiver burden measures

*HRQoL* was assessed for both children with CP and their primary caregivers using preference-based instruments aligned with economic evaluation standards. For children, the *proxy version of the EQ-5D-Y* [27] was completed by caregivers at baseline, 6 months, and 12 months. For caregivers, their own HRQoL was measured using the *EQ-5D-5L* [31], administered at the same three time points. Both instruments evaluate five core health dimensions: mobility, self-care, usual activities, pain/discomfort, and anxiety/depression rated at three levels EQ-5D-Y and five levels EQ-5D-5L of severity, respectively.

The EQ-5D-Y and EQ-5D-5L instruments generate *preference-weighted health utility values* derived from population-based tariffs [32, 33]. These *utilities represent HRQoL* on a cardinal scale anchored at *0 (equivalent to death) and 1 (full health)*, with negative values permitted for health states considered worse than death [27]. *Health utility values* constitute the fundamental *input* for the calculation of quality-adjusted life years *(QALYs)*, which *combine utility with time* to integrate morbidity and survival into a single metric widely used in economic evaluation and health technology assessment [34].

In the present study, all cost estimates corresponded to a 12-month period per participant, and health utility values were observed within the same annual analytic framework. No extrapolation beyond the observed 12-month horizon or long-term modelling of QALY accumulation was performed. Utility scores were derived using the respective Spanish value sets. Individual QALYs were estimated using the area-under-the-curve (AUC) method, assuming linear interpolation between baseline, 6-month, and 12-month assessments. This approach generated continuous time-weighted utility profiles for both children and caregivers within the defined annual study period, facilitating their incorporation into individual-level predictive models [23, 24].

Caregiver burden was also measured using the *Zarit Burden Interview (ZBI)*, a validated 22-item instrument capturing subjective burden across emotional, physical, social, and financial domains. Responses were recorded on a 5-point Likert scale, yielding total scores from 0 to 88, and categorized as follows: 0–20 (none to mild), 21–40 (mild to moderate), 41–60 (moderate to severe), and 61–88 (severe burden). The ZBI has demonstrated high internal consistency (Cronbach’s alpha = 0.93) and test-retest reliability (0.89), and offers a complementary latent measure of caregiver strain with relevance for both quality of life and resource use [35].

#### Classification of cost components by perspective and source

A *societal perspective* was adopted to estimate the total economic burden of CP, incorporating *costs incurred by the healthcare system*, the *public sector*, and *families*. Individual-level costs were *calculated* using a *bottom-up* approach and categorized into six components: (1) total disease-related costs; (2) direct healthcare costs; (3) informal care costs; (4) public disability-related subsidies; (5) out-of-pocket (OOP) family expenses, *including the costs of intensive and emerging rehabilitation therapies* used alongside public healthcare services; and (6) total societal costs, defined as the sum of all components. All costs are reported in 2023 euros. Aggregate population-level expenditures were estimated by summing individual-level costs across the sample [36].

*Direct medical costs* included healthcare services covered by the public system, such as general practitioner visits, specialist consultations, diagnostic and follow-up tests, hospital admissions, emergency care, ambulance transport, prescription drugs, and standard treatments. *Unit costs were based on Spanish pharmacoeconomic guidelines*, primarily drawn from the Analytical Accounting Department of the HUN. *Drug prices were sourced from the Navarra Health Service Pharmacy Department*, while *outpatient visits* were adjusted using specialty-specific coefficients *according to regional hospital tariffs* [37, 38].

*Government expenditures* included disability-related financial benefits, funding for *special education*,* and subsidies* for assistive technologies such as *mobility or communication aids*. These were *valued* using *administrative budget data* and individual allocations provided by regional authorities, including the Department of Social Rights and the Special Education Resource Centre of Navarra (CREENA). *Family-incurred costs* encompassed OOP spending on *Intensive and Emerging Rehabilitation Therapies (Physiotherapy*,* speech therapy*,* occupational therapy*,* homeopathy hippotherapy*,* Therasuit*,* Peto Method)*, non-reimbursed assistive devices, transport to healthcare or educational facilities, home modifications, and *productivity losses due to informal caregiving*. Productivity losses were *valued using the replacement cost method* [39, 40]. Transportation costs were estimated based on reported travel frequency and mode.

#### Missing data and data completeness

Proxy EQ-5D-Y assessments for children and self-reported EQ-5D-5L assessments by primary caregivers at baseline and at 6 months were complete. Cost data and predictor variables were fully recorded thanks to a multisectoral data collection approach using clinical records, administrative databases, analytical accounting, and caregiver reports.

The only missing data corresponded to 5 out of 148 primary caregivers (3.4%) in the EQ-5D-5L assessment at 12 months, attributable to time constraints in the intensive/emerging rehabilitation therapy (IERT) subgroup. Children’s EQ-5D-Y assessments remained fully recorded at 12 months. Since the study was a one-year, cross-sectional design and reached its planned completion, these caregiver data could not be collected at the final visit.

To preserve the robustness of the analysis, the missing 12-month EQ-5D-5L values for these 5 caregivers were *imputed using their own 6-month measurements*, a conservative approach consistent with best practices in health economics and outcomes research [41]. All models otherwise employed a complete-case approach, ensuring analytical integrity without introducing unverifiable assumptions.

### Statistical analysis

#### Cost modelling and its predictors

**Fig. 1:**
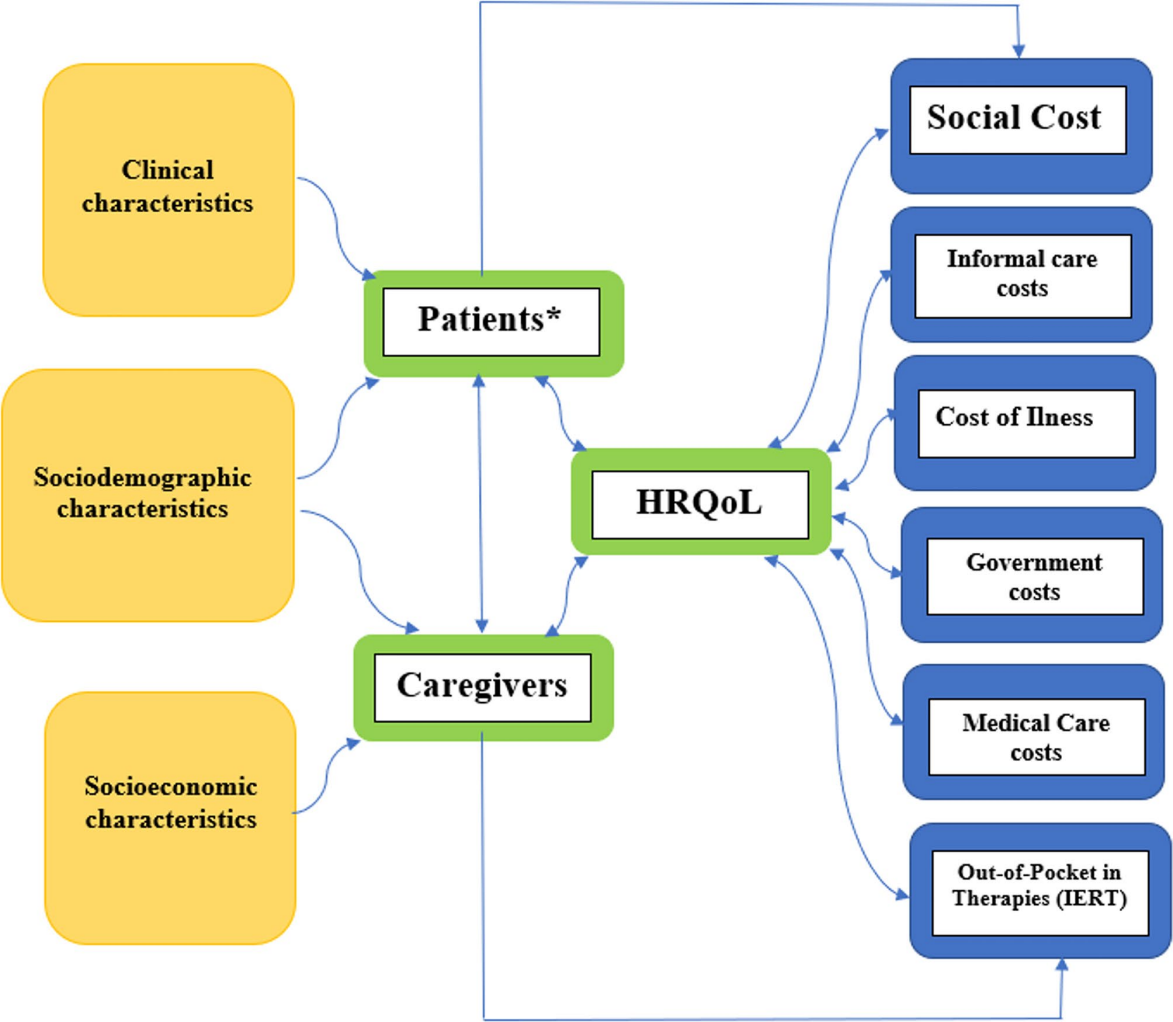
Graphical Representation of Cost and Health-Related Quality of Life Models incorporating individual-level characteristics of children with cerebral palsy and their Caregivers. *IERT: Cost of Intensive and Emerging Rehabilitation Therapies. Government Cost: included disability-related financial benefits, funding for special education, and subsidies for assistive technologies such as mobility or communication aids

Cost data were collected over a one-year observation period following each participant’s enrollment in the Epidemiological Registry of the HUN. Annual costs were calculated by summing all relevant expenditures incurred during this period. Separate statistical models were constructed for each of six predefined cost categories: (1) informal care costs, (2) social costs, (3) total cost of illness (COI), (4) government/public costs, (5) medical care costs, and (6) out-of-pocket (OOP) family costs associated with Intensive and Emerging Rehabilitation Therapies. All cost estimates were generated from a societal perspective, and the overall framework is schematically depicted in Fig. 1.

Candidate predictors for all models included a broad set of baseline covariates, grouped into four domains:

- *Sociodemographic variables*: child’s and caregiver’s age and sex, geographic origin, and residential setting;
- *Socioeconomic variables*: caregiver’s education level and employment status, marital status, household income, social class, and school support;
- *Clinical variables*: CP types, functional classification systems (GMFCS, VSS, VFCS, MACS, EDACS, CFCS, BFMF), cerebral palsy etiology, neuroimaging findings (MRICS), and overall impairment index;
- *Health-related and caregiver experience variables*: caregiver burden (Zarit Burden Interview) and quality-adjusted life years (QALYs) for both children and caregivers.

To address the right-skewed nature of most cost outcomes, generalized linear models (GLMs) were used as the primary analytical approach [19, 42]. For OOP expenditures on Intensive and Emerging Rehabilitation Therapies, which included a large proportion of zero values a two-part model was applied: (1) a logistic regression estimated the probability of incurring any cost, and (2) a GLM estimated the amount spent conditional on positive expenditure [19]. For the second part of the two-part model, three model specifications were compared using combinations of Gaussian and Gamma distributions with identity and log link functions. Final model selection was guided by goodness-of-fit criteria, predictive accuracy, and parsimony.

#### Modelling health-related quality of life (HRQoL) and caregiver burden

Child health-related quality of life (HRQoL) and caregiver subjective burden were modelled using the same set of sociodemographic, socioeconomic, and clinical covariates applied in the cost models. In addition, mutually exclusive cost subcategories were included as predictors: informal care costs, medical care costs, government transfers/subsidies, and out-of-pocket expenses (specifically those related to Intensive and Emerging Rehabilitation Therapies), as illustrated in Fig. 1. Broader aggregate categories such as total cost of illness or social cost were not included, as they encompass the cost components already specified in the model. HRQoL outcomes for both children (QALYs) and caregivers were estimated using ordinary least squares (OLS) and censored regression (Tobit) models, as appropriate. The same modelling strategies were applied to assess subjective caregiver burden using Zarit Burden Interview scores.

#### Variable selection, goodness-of-fit and parsimony

Final model selection was guided by three complementary criteria: (i) parsimony and goodness-of-fit assessed through the Akaike Information Criterion (AIC), (ii) statistical significance of predictors in HRQoL and caregiver burden models using a conservative threshold (*p* < 0.01), and (iii) predictive accuracy and internal validation procedures described in Section “Predictive accuracy, internal validation and final model selection”.

For the *cost models*, we employed a full‑subset selection strategy ($\:{2}^{p}$), where p denotes the number of candidate covariates and all possible combinations of these variables were evaluated [43]. The final specification for each cost outcome was chosen as the model with the lowest AIC, thus prioritising parsimony while maintaining adequate goodness‑of‑fit. For *HRQoL and caregiver burden models*, we applied a *hybrid stepwise procedure (forward selection and backward elimination)* with a conservative significance threshold of *p* < 0.01 to control for multiple comparisons and reduce overfitting [43]. Together, these procedures define the criteria used to determine the final model specifications.

#### Predictive accuracy, internal validation and final model selection

To assess predictive accuracy and model generalisability, we used two internal validation strategies, each applied to a specific set of models according to the nature of their outcomes. For models: (1) out‑of‑pocket costs for intensive and emerging rehabilitation therapies (two‑part model), (2) health‑related quality of life (QALYs) in children and caregivers, and (3) caregiver burden (Zarit scores)—we performed *repeated 10‑fold cross‑validation*. The sample was randomly partitioned into ten folds; in each iteration, eight folds were used for model training and two for validation, ensuring all observations served as validation data cross folds. This process was repeated across several random splits to increase robustness. *Predictive accuracy* in these models was summarised using mean error *(ME)*, mean absolute error *(MAE)*, and root mean square error *(RMSE)*, and the final specification for each outcome was chosen as the model with the lowest combination of these error metrics.

*For the remaining cost outcomes*—Informal Care, Social Costs, Cost of Illness, Government Costs, and Medical Care Costs—we focused instead on *parameter uncertainty* and coefficient stability. We applied *non‑parametric bootstrap resampling* (1,000 iterations) to obtain bias‑corrected and accelerated *(BCa)* 95% confidence intervals, which adjust for skewness and bias in the empirical distribution and are particularly suitable for non‑normal, skewed outcomes such as those modelled with Gamma distributions and log links. This approach allowed us to evaluate the stability of coefficient estimates and to identify predictors that were especially sensitive to sampling variability.

## Results

### Descriptive analysis

Among the different components of the social cost associated with CP, out-of-pocket (OOP) expenditures played a significant role in shaping the overall economic burden and were closely linked to variations in HRQoL. In this study, two distinct subgroups emerged based on the presence or absence of disease-related OOP healthcare expenditures: one group reported significant costs associated with Intensive and Emerging Rehabilitation Therapies, primarily accessed through the private healthcare sector, while the other group incurred no such expenses. Importantly, individuals in the OOP group exhibited a greater variability in HRQoL outcomes, suggesting a potential association between these additional costs and perceived health needs or benefits [10].

Table S1(Supplementary Material) presents the baseline characteristics of the 148 individuals with cerebral palsy (CP) and their caregivers, disaggregated by the presence or absence of out-of-pocket (OOP) healthcare expenditures. The mean age of children was 9.78 years (SD = 4.25), and 53% were male. Caregivers had a mean age of 42.97 years (SD = 6.25), and 66% were female. Most caregivers were married (72%), and 73% had higher education. Primary education was more common among those without OOP expenditures (16% vs. 6.5%). Differences by ethnic origin were also notable: 77% of caregivers with OOP expenditures were Caucasian, compared to 29% among those without. Additionally, nearly half of caregivers (49%) reported having quit or lost a job due to the child’s disability.

In terms of *functional status*, greater severity was observed in the OOP group, particularly in GMFCS level V (34% vs. 14%) and MACS level V (26% vs. 13%). Likewise, a higher proportion of individuals with OOP expenditures were classified in the most severe category of the impairment index (49% vs. 29%). In contrast, milder functional limitations such as GMFCS I or BFMF I were more prevalent among those without OOP expenses. The most frequent CP type was spastic (79%), especially among those without OOP expenditures (89%). Lastly, geographic accessibility to health services differed markedly: 60% of children with OOP costs reported easy access to care, whereas 30% of those without OOP expenditures lived in areas with no local health services available [44].

### Individual-level prediction of annual costs in pediatric cerebral palsy

#### Descriptive overview of annual costs

The estimated average annual societal cost per child with CP was €102,135, underscoring the broad and heterogeneous economic burden. Direct healthcare costs averaged €3,801 (3.72% of the total), whereas the largest component was caregiver productivity loss (€60,638; 59.37%), followed by special education services (€8,932; 8.75%). OOP expenditures averaged €7,041 (6.89%), predominantly for Intensive and Emerging Rehabilitation Therapies, which were utilized by 64% of families [11]. Children with severe motor impairments (GMFCS III–V) incurred nearly double the annual costs compared to those with mild impairments (GMFCS I–II), with a cost ratio of 1.96 (95% CI: 1.92–2.01), highlighting the economic implications of functional severity [36].

#### Models predicting CP-related annual costs by component

Using generalized linear models (GLM) with gamma distribution and log link, five predefined cost categories were analysed: (1) Informal care, (2) Social costs, (3) Costs of illness, (4) Government costs, and (5) Medical care costs (See Table 1). OOP costs were modelled using two-part models: a logistic regression (probability of incurring any cost) followed by conditional GLMs with identity and log link functions (See Table 2).

Across all categories, multivariable models identified GMFCS III–V as the most robust predictors of higher costs (*p* < 0.001), especially for disease-related, social, and government-attributed expenditures. Informal care costs were significantly associated with caregiver burden (Zarit score), child HRQoL (z_QALYsC), and child sex and ethnicity—male or Latin American children incurred lower caregiving costs (*p* < 0.05).

**Table 1:** Annual costs (€) by components of paediatric cerebral palsy: Generalised linear models with Gamma distribution and log link function

Predictor	Model 1: Informal Care Cost	Model 2: Social Cost	Model 3: Cost of Illness	Model 4: Government Costs	Model 5: Medical Care Costs
**(Intercept)**	10.638 (0.184) ***	11.021 (0.102) ***	9.842 (0.069) ***	8.899 (0.144) ***	8.339 (0.048) ***
SEXC: Male	-0.085 (0.049) ·	-0.035 (0.026)	—	—	-0.087 (0.047) ·
SEXP: Male	—	-0.041 (0.029)	-0.062 (0.030) *	—	-0.097 (0.044) *
GOI: Caucasian	-0.104 (0.068)	—	0.056 (0.051)	—	—
GOI: Latin American	-0.181 (0.088) *	—	0.155 (0.058) **	—	—
CFCS II	-0.142 (0.071) ·	—	0.191 (0.070) **	—	—
CFCS III	-0.031 (0.071)	—	0.234 (0.071) **	—	—
CFCS IV	0.114 (0.079)	—	0.238 (0.100) *	—	—
CFCS V	-0.036 (0.084)	—	0.197 (0.110) ·	—	—
GMFCS II	—	0.051 (0.047)	0.329 (0.049) ***	0.307 (0.067) ***	—
GMFCS III	—	0.331 (0.049) ***	0.788 (0.057) ***	0.726 (0.072) ***	—
GMFCS IV	—	0.598 (0.071) ***	1.145 (0.091) ***	0.934 (0.104) ***	—
GMFCS V	—	0.549 (0.071) ***	1.186 (0.108) ***	1.055 (0.100) ***	—
VSS II	—	—	-0.187 (0.069) **	—	—
VSS III	—	—	-0.151 (0.084) ·	—	—
VSS IV	—	—	-0.111 (0.093)	—	—
VFCS II	—	—	0.031 (0.053)	—	—
VFCS III	—	—	0.109 (0.063) ·	—	—
VFCS IV	—	—	-0.092 (0.070)	—	—
VFCS V	—	—	-0.085 (0.087)	—	—
EDACS II	—	—	-0.002 (0.073)	—	-0.0003 (0.063)
EDACS III	—	—	-0.102 (0.079)	—	-0.157 (0.070) *
EDACS IV	—	—	-0.077 (0.099)	—	-0.069 (0.079)
EDACS V	—	—	0.101 (0.119)	—	0.036 (0.077)
ETIOL: Postnatal	-0.053 (0.078)	0.009 (0.047)	0.072 (0.053)	—	—
ETIOL: Prenatal	0.023 (0.071)	-0.003 (0.042)	-0.085 (0.034) *	—	—
ETIOL: Unknown	-0.618 (0.158) ***	-0.342 (0.088) ***	0.084 (0.085)	—	—
MRI Pattern B	-0.059 (0.093)	-0.031 (0.054)	—	—	—
MRI Pattern C	-0.042 (0.084)	0.005 (0.050)	—	—	—
MRI Pattern D	0.213 (0.082) * ·	0.072 (0.049)	—	—	—
MRI Pattern E	0.262 (0.106) *	0.181 (0.064) **	—	—	—
CIVISTAT: Married	—	—	—	-0.020 (0.061)	—
CIVISTAT: Single	—	—	—	0.121 (0.075) ·	—
School: Normal + Support	—	—	-0.015 (0.045)	—	—
School: Special center	—	—	-0.087 (0.063)	—	—
School: Special class	—	—	0.039 (0.053)	—	—
Zarit Score	0.007 (0.002) **	0.003 (0.001) ·	—	-0.001 (0.002)	—
z_QALYsC	-0.131 (0.034) ***	—	0.143 (0.029) ***	—	—
z_QALYsA	—	—	-0.037 (0.026)	—	-0.094 (0.027) ***
z_AGEC	—	—	-0.033 (0.016) *	—	—
z_AGEP	—	-0.023 (0.013) ·	—	—	—
z_HOUSEINCY	—	—	0.038 (0.018) *	—	—

Note: Estimates are presented as coefficients with standard errors in parentheses. Statistical significance is indicated as follows

*** *p* < 0.001; ** *p* < 0.01; * *p* < 0.05; - *p* < 0.10; no symbol indicates *p* ≥ 0.10. The variables are defined as follows: *(Intercept)* refers to the model intercept. SEXC and SEXP indicate male sex of the caregiver and the child, respectively. GOI: Caucasian and GOI: Latin American refer to the child’s geographical origin. CFCS II–V indicate levels II to V of the Communication Function Classification System. GMFCS II–V refer to levels of the Gross Motor Function Classification System. VSS II–IV indicate levels of the Viking Speech Scale, and VFCS II–V denote levels of the Visual Function Classification Scale. EDACS II–V correspond to levels of the Eating and Drinking Ability Classification System. ETIOL refers to the etiology of cerebral palsy: postnatal, prenatal, or unknown. MRI Pattern B–E indicate classification levels of magnetic resonance imaging findings. CIVISTAT: Married/Single refers to the caregiver’s civil status. School: Normal + Support, Special center, and Special class indicate the type of school support received. Zarit Score, Zarit caregiver burden score. Prefix z_ indicates that variables were standardized. z_QALYsC and z_QALYsA represent standardized QALYs gained by the child and caregiver, respectively. z_AGEC and z_AGEP refer to standardized age of the caregiver and child z_HOUSEINCY corresponds to standardized annual household income

Other clinical factors such as communication limitations (CFCS) and speech limitations (VSS) are also associated with increases in the total cost of illness. Caregiver burden (Zarit) and lower quality of life for the child (QALYsC) predict higher informal care costs, reflecting the *direct impact of family well-being* on the demand for unpaid resources.

In addition, sociodemographic variables such as caregiver gender and the child’s Latin American origin are significantly related to costs, although with divergent patterns: children of Latin American origin have lower informal care costs but higher total costs, which could reflect differences in access to or use of formal versus informal resources. Finally, some findings, such as the association between higher QoL for children and higher total social costs, suggest that families with greater therapeutic commitment incur higher expenses, possibly linked to the use of unconventional therapies. These results reinforce the importance of incorporating both clinical complexity and social determinants into economic evaluation models for a more equitable and family-centred allocation of resources.

#### Two-part model for out-of-pocket expenses related to intensive and emergent rehabilitation therapies

The two-part model assessing household spending on Intensive and Emerging Rehabilitation Therapies revealed a consistent and marked cost gradient shaped by functional severity. *Results from both stages of the model are presented in* Table 2. *In the first part of model*, which estimated the probability of incurring any out-of-pocket costs, none of the included predictors reached statistical significance. However, positive coefficients were observed for higher levels of gross motor impairment (GMFCS III to V) and for child-reported quality of life (z_QALYsC), suggesting possible trends toward increased uptake of these therapies in these groups. In contrast, sociodemographic factors such as social class, type of school, or geographic access to public services were not significantly associated with the use of these interventions. This may indicate that, at least in this sample, initial access is more closely driven by clinical characteristics than by structural barriers.

**Table 2:** Annual out-of-pocket cost models for external health care expenditures on intensive and emerging rehabilitation therapies (€): two-part model (part 1: logistic regression; part 2: generalised linear models with gaussian and gamma distributions and identity/log link functions)

Predictor	Part 1: Likelihood of incurring cost	Part 2: Cost, if any incurred		
		Part 2 A: GLM Gaussian (Identity)	Part 2B: GLM Gaussian (Log)	Part 2 C: GLM Gamma (Log)
**(Intercept)**	-354.5 (322.9)	3936.5 (2093.2) ·	8.9452 (0.234) ***	8.199 (0.247) ***
SEXPM (Ref: F)	-34.90 (29.95)	—	—	—
GMFCS II (Ref: I)	61.58 (55.92)	4998.8 (1456.6) ***	0.647 (0.219) **	0.698 (0.139) ***
GMFCS III (Ref: I)	213.27 (184.27)	8178.8 (1550.6) ***	0.912 (0.208) ***	1.024 (0.151) ***
GMFCS IV (Ref: I)	735.72 (6829.08)	14016.7 (2357.9) ***	1.398 (0.254) ***	1.545 (0.226) ***
GMFCS V (Ref: I)	649.27 (542.16)	12046.8 (2650.1) ***	1.190 (0.248) ***	1.412 (0.254) ***
GOI (Caucasian. Ref: Arabic)	34.650 (3629)	—	—	—
GOI (Latino. Ref: Arabic))	169.06 (23411.45)	—	—	—
LeaveJobCPYes (Ref: No)	—	-1047.5(647.5)	0.119 (0.057) *	—
Social Class: Low (Ref: High)	71.41 (66.31)	-1515.4 (1501.6)	-0.192 (0.144)	-0.296 (0.140) *
Social Class: Lower-Middle	28.49 (1561.45)	785.9 (1330.2)	0.100 (0.120)	0.013 (0.120)
Social Class: Middle	54.77 (55.48)	1433.5 (1214.4)	0.135 (0.109)	0.064 (0.113)
School: Normal with support	—	-3852.6 (1084.3) ***	-0.338 (0.100) **	-0.380 (0.106) ***
School: Special center (Ref: Normal)	—	-3853.2 (1370.9) **	-0.321 (0.118) **	-0.222 (0.166)
School: Special class	—	-2198.4 (1164.6) ·	-0.194 (0.107) ·	-0.084 (0.136)
MRICSB (Ref: A)	9.057 (38.86)	—	—	—
MRICSC (Ref: A)	54.58 (54.83)	—	—	—
MRICSD (Ref: A)	4.93 (39.11)	—	—	—
MRICSE (Ref: A)	143.91 (126.06)	—	—	—
VFCSII (Ref: I)	—	-824.3 (949.1)	-0.086 (0.088)	—
VFCSIII (Ref: I)	—	-1652.9 (1075.1)	-0.194 (0.091) *	—
VFCSIV (Ref: I)	—	2023.8 (1529.4)	0.147 (0.122)	—
VFCSV (Ref: I)	—	-141.1 (1355.6)	-0.037 (0.114)	—
RESID: Limited access to health services (Ref: Full Access)	—	1868.2 (757.8) *	0.138 (0.065) *	0.205 (0.071) **
RESID: No local access to health services	—	2649.6 (1015.5) *	0.266 (0.086) **	0.293 (0.100) **
Zarith_Score	—	—	-0.006 (0.004)	—
Impairment Index: Low (Ref: Severe)	—	—	—	0.236 (0.163)
Impairment Index: Moderate	—	—	—	0.027 (0.133)
z_AGEP	-18.66(18.49)	—	—	—
z_QALYsC (Child HRQoL, standardized)	271.85 (229.51)	1194.8 (870.2)	—	0.132 (0.084)

Note: Estimates are presented as coefficients with standard errors in parentheses. Statistical significance is indicated as follows:

*** *p* < 0.001; ** *p* < 0.01; * *p* < 0.05; - *p* < 0.10; no symbol indicates *p* ≥ 0.10. The variables are defined as follows: *(Intercept)* is the model intercept. SEXPM indicates the sex of the primary caregiver (1 = male). GMFCS II–V refer to levels of the Gross Motor Function Classification System. GOI, represents the group of origin or immigration background. LeaveJobCPYes identifies whether the caregiver had to leave a job due to the child’s cerebral palsy. Social Class, indicates the caregiver’s self-reported socioeconomic status. School, describes the type of educational support the child receives. MRICS, refer to categories of the MRI Classification System for cerebral palsy. VFCS, indicate levels of the Visual Function Classification System. RESID: Limited and No local access to health services refer to residential areas with restricted access to public healthcare. Zarith_Score represents the caregiver burden score based on the Zarit Burden Interview. Impairment Index, reflects composite levels of functional impairment. z_AGEP and z_QALYsC represent standardized scores for caregiver age and child health-related quality of life, respectively

In the *second part of the model*, the amount of out-of-pocket spending was consistently and significantly associated with the level of gross motor impairment (GMFCS), with higher levels predicting greater expenditures across all model specifications. Attending regular schools with support or specialized education centers was linked to significantly lower costs (*p* < 0.01), suggesting that educational environments may play a role in shaping care trajectories. Living in areas with limited or no access to public healthcare services significantly increased spending (*p* < 0.01), indicating a compensatory use of private or non-conventional care. Belonging to a low (vs. high) social class was inversely associated with expenditures in the Gamma model (*p* = 0.038), possibly reflecting affordability barriers or constrained access.

Taken together, these findings suggest that expenditures on intensive and emerging rehabilitation therapies are not random but instead reflect a confluence of clinical need, perceived therapeutic benefit, and access-related inequities. The results highlight the importance of policy measures that not only address unmet rehabilitative needs among children with complex disabilities, but also reduce structural and economic constraints that limit families’ ability to access and sustain hybrid care pathways.

### Health-related quality of life (HRQoL) prediction models

To facilitate dyadic comparison, we present prediction models of HRQoL for children with cerebral palsy and their primary caregivers in a single table, highlighting shared and distinct predictors across both populations. This approach aligns with the individual-level framework adopted throughout the study. Child HRQoL (QALYsC) was assessed using the proxy version of the EQ-5D-Y completed by the primary caregiver, while caregiver HRQoL (QALYsA) was self-reported using the EQ-5D-5L instrument. Multivariable linear (OLS) and censored (Tobit) regression models were estimated separately for both outcomes. Table 3 summarizes the estimated coefficients, standard errors, and significance levels for key predictors across models.

**Table 3:** Prediction models of health-related quality of life in children with cerebral palsy and their primary caregivers: OLS and tobit estimates for QALYs

Predictor	Children with CP		Primary Caregivers	
	OLS1 Model	Tobit1 Model	OLS2 Model	Tobit2 Model
**(Intercept)**	0.495 (0.085) ***	0.500 (0.080) ***	0.857 (0.053) ***	0.857 (0.048) ***
AGE (Child / Adult)	0.0074 (0.0016) ***	0.0075 (0.0016) ***	0.0040 (0.0016) *	0.0040 (0.0014) **
CPT: Spastic	-0.069 (0.026) **	-0.067 (0.025) **	—	—
GMFCS II	-0.094 (0.038) *	-0.104 (0.036) **	-0.0032 (0.022)	-0.0032 (0.020)
GMFCS III	-0.171 (0.042) ***	-0.181 (0.040) ***	-0.063 (0.025) *	-0.063 (0.022) **
GMFCS IV	-0.600 (0.061) ***	-0.611 (0.058) ***	-0.198 (0.035) ***	-0.198 (0.032) ***
GMFCS V	-0.633 (0.062) ***	-0.641 (0.059) ***	-0.218 (0.037) ***	-0.218 (0.033) ***
CFCS III	-0.114 (0.033) ***	-0.117 (0.031) ***	—	—
CFCS V	-0.071 (0.041) ·	-0.074 (0.039) ·	—	—
Zarit poly (1st)	-0.334 (0.230)	-0.338 (0.217)	-0.389 (0.133) **	-0.389 (0.120) **
Zarit poly (2nd)	0.211 (0.131)	0.210 (0.124) ·	-0.0066 (0.081)	-0.0066 (0.074)
Zarit poly (3rd)	0.206 (0.130)	0.208 (0.123) ·	-0.149 (0.077) ·	-0.149 (0.070) *
ETIOL: Unknown	-0.176 (0.057) **	-0.181 (0.054) ***	—	—
Tot_CostTTCPAL	0.0000199 (0.0000019) ***	0.0000202 (0.0000018) ***	—	—
Income (z_HOUSEINCY)	—	—	8.95e-07 (3.92e-07) *	8.95e-07 (3.55e-07) *
Education: Tertiary	—	—	-0.061 (0.033) ·	-0.061 (0.030) *
Civil Status: Married	—	—	-0.035 (0.019) ·	-0.035 (0.017) *
School: Special class	—	—	-0.043 (0.020) *	-0.043 (0.018) *
BFMF IIIb	—	—	-0.047 (0.025) ·	-0.047 (0.022) *
RESID: No local access	-0.037 (0.026)	-0.0387 (0.024)	0.0369 (0.016) *	0.0369 (0.014) **

Note: Estimates are presented as coefficients with standard errors in parentheses. Statistical significance is indicated as follows: *** *p* < 0.001; ** *p* < 0.01; * *p* < 0.05; - *p* < 0.10; no symbol indicates *p* ≥ 0.10. Variable definitions: (Intercept) are the model intercept. AGE (Child / Adult) indicates the age group of the children with CP. CPT: Spastic refers to the spastic type of cerebral palsy. GMFCS II–V correspond to levels II to V of the Gross Motor Function Classification System. CFCS III and CFCS V refer to levels III and V of the Communication Function Classification System. Zarit poly (1st–3rd) denote the first through third degree polynomial terms of the Zarit Caregiver Burden Scale. ETIOL: Unknown indicates unknown etiology. Tot_CostTTCPAL represents out-of-pocket costs for intensive and emerging rehabilitation therapies. Income (z_HOUSEINCY) is the standardized household income. Education: Tertiarys indicates caregiver’s highest educational attainment at tertiary level. Civil Status: Married denotes marital status. School: Special class indicates special education support received by the children. BFMF IIIb corresponds to level IIb in the fine manual motor function classification. RESID: No local access identifies residence with no local access to health services

HRQoL prediction models, estimated using both OLS and Tobit regressions for children and caregivers, revealed distinct yet complementary patterns in the factors associated with reported health utilities. Among children with CP, higher levels of motor impairment (GMFCS III–V) were systematically associated with lower HRQoL in both linear and censored models (*p* < 0.001), with reductions of up to 0.63 QALY units compared to children with milder impairments. Additionally, the spastic clinical phenotype (*p* < 0.01), communicative function limitations (CFCS III, *p* < 0.001), and unknown etiology (*p* = 0.003) were negatively associated with child wellbeing. Notably, annual expenditures on Intensive and Emerging Rehabilitation Therapies (Tot_CostTTCPAL) were positively associated with child HRQoL (*p* < 0.001), which may reflect either perceived benefit or greater family engagement in treatment. Child age also had a positive effect (*p* < 0.001), suggesting possible functional adaptation or improvement over time.

In the case of primary caregivers, the impact of caregiver burden (Zarit Score) was clear: the linear component showed a strong negative association with caregiver QALYs (*p* < 0.01 in both models), and the cubic term was also significant in the Tobit model (*p* = 0.032), indicating a nonlinear relationship in how burden affects perceived wellbeing. Child motor severity remained a key determinant of caregiver HRQoL, with significant effects for GMFCS levels III–V (*p* < 0.001). Sociodemographic factors such as lower household income (*p* = 0.012), higher educational attainment (*p* = 0.038), and being married (*p* = 0.045) were also associated with lower HRQoL, potentially reflecting a greater perceived burden or role conflict in reconciling caregiving with other responsibilities. Interestingly, living in areas without local access to healthcare services was paradoxically associated with higher caregiver HRQoL (*p* = 0.009), which may reflect adaptive resilience mechanisms in settings with reduced institutional support.

Taken together, these results underscore the importance of accounting for both the child’s clinical severity and the social and contextual determinants surrounding the family unit when evaluating health impacts and designing wellbeing-centred interventions. Incorporating child- and caregiver-specific HRQoL measures, as done in this study, enables a more comprehensive assessment of health value from an individual and family-centred perspective.

### Subjective caregiver burden

Caregiver burden, measured using the Zarit Burden Interview, was significantly associated with the child’s motor severity, caregiver health status, and socioeconomic conditions (Table 4). Both OLS and Tobit models showed that greater functional impairment (GMFCS II–V) was consistently linked to higher burden scores (*p* < 0.001), confirming the cumulative effect of physical dependency. Caregiver HRQoL (QALYsA) was a strong negative predictor (*p* < 0.001), reinforcing the close link between subjective wellbeing and perceived strain.

Additional factors included employment (marginally protective), lower-middle social class (associated with higher burden), and increased informal care costs (*p* < 0.01), reflecting the resource intensity of caregiving. Out-of-pocket spending on non-standard therapies also showed a marginal association with greater burden (*p* = 0.054). Surprisingly, caregivers in areas with limited health service access reported lower burden (*p* < 0.05), potentially reflecting resilience or adaptation in low-support environments. Overall, these results underscore caregiver burden as a critical outcome in economic evaluations and family-centred planning. Incorporating validated measures like the Zarit scale enhances understanding of the broader psychosocial and financial impact of paediatric CP, informing more equitable policy and resource distribution.

**Table 4:** Prediction models for caregiver burden (Zarit score): OLS and tobit estimates

Predictor	OLS Model	Tobit Model
**(Intercept)**	70.97 (8.67) ***	60.80 (6.96) ***
SEX: Male caregiver	1.80 (1.14)	1.79 (1.08) ·
GMFCS II	9.81 (1.95) ***	10.38 (1.77) ***
GMFCS III	8.43 (2.80) **	10.75 (2.30) ***
GMFCS IV	14.94 (5.21) **	22.73 (3.23) ***
GMFCS V	13.69 (5.60) *	23.31 (3.43) ***
Caregiver employed (LevJobCP)	-2.24 (1.15) ·	-2.06 (1.09) ·
Caregiver HRQoL (QALYsA)	-27.37 (7.76) ***	-25.60 (7.45) ***
Social Class: Low	-0.02 (2.33)	1.62 (2.19)
Social Class: Lower-Middle	3.78 (2.09) ·	4.81 (1.98) *
Social Class: Middle	2.30 (2.01)	3.38 (1.88) ·
Child HRQoL (QALYsC)	-5.99 (4.58)	—
Government-attributed costs (Tot_CostGov)	-0.00031 (0.00021)	-0.00033 (0.00020) ·
CFCS III	-6.23 (2.12) **	—
Out-of-pocket IERT costs (Tot_CostTTCPAL)	0.00030 (0.00016) ·	—
Informal care costs (CosT_Ycare)	0.00011 (0.00004) **	0.00010 (0.00004) **
RESID: Limited access	-3.07 (1.41) *	-2.99 (1.34) *
RESID: No local access	0.76 (1.59)	0.13 (1.50)
IMP_INDEX: Low	-4.74 (2.43) ·	—
IMP_INDEX: Moderate	-2.29 (2.00)	—

Note: Estimates are presented as coefficients with standard errors in parentheses. Statistical significance is indicated as follows: *** *p* < 0.001; ** *p* < 0.01; * *p* < 0.05; - *p* < 0.10; no symbol indicates *p* ≥ 0.10. IERT: Intensive and Emerging Rehabilitation Therapies. Variable definitions: SEX: Male caregiver indicates the sex of the caregiver as male. GMFCS II–V correspond to levels II to V of the Gross Motor Function Classification System. Caregiver employed (LevJobCP) refers to whether the primary caregiver left their job due to having a family member with cerebral palsy. Caregiver HRQoL (QALYsA) denotes the health-related quality of life of the caregiver, measured in quality-adjusted life years. Social Class categories indicate socioeconomic status: Low, Lower-Middle, and Middle. Child HRQoL (QALYsC) refers to the child’s health-related quality of life, measured in quality-adjusted life years. Government-attributed costs (Tot_CostGov) correspond to costs borne by government entities. CFCS III is level III of the Communication Function Classification System. Out-of-pocket IERT costs (Tot_CostTTCPAL) are the direct expenses paid by families for intensive and emerging rehabilitation therapies. Informal care costs (CosT_Ycare) represent the economic value of unpaid caregiving. RESID: Limited access and RESID: No local access describes residence areas with limited or no access to health services, respectively. IMP_INDEX: Low and IMP_INDEX: Moderate indicate levels of impairment severity

### Model validation and predictive accuracy

Cross-validation analyses supported the reliability and predictive accuracy of all models across key outcomes (Table 5). For out-of-pocket expenditures related to Intensive and Emerging Rehabilitation Therapies, the best performance was achieved using a generalized linear model with Gaussian distribution and log link, which minimized prediction errors and bias.

Regarding HRQoL, ordinary least squares (OLS) models consistently yielded the lowest prediction errors for both children and caregivers, with negligible differences compared to Tobit models. Similarly, caregiver burden (Zarit score) was well predicted by both OLS and Tobit specifications, with only marginal gains in fit using the censored model.

**Table 5:** Cross-validation performance metrics (RMSE, MAE, ME) by outcome and model specification

Outcome / Model Component	Model	RMSE	MAE	ME
**Out-of-Pocket IERT Costs – Part 2 of Two-Part Model**				
	Gaussian Identity	3143.34	2614.53	-22.43
	Gaussian Log	**3146.97**	**2586.79**	**-20.73**
	Gamma Log	3208.53	2637.96	-65.14
**Health-Related Quality of Life (HRQoL)**				
QALYs Child (proxy-reported)	OLS	**0.136**	**0.110**	**-0.0018**
	Tobit	0.137	0.112	-0.0047
QALYs Caregiver	OLS	**0.0731**	**0.0551**	**-0.0011**
	Tobit	0.0731	0.0551	-0.0011
**Caregiver Burden (Zarit Score)**				
	OLS	7.023	5.551	0.0354
	Tobit	**7.023**	**5.544**	**0.0193**

Note: RMSE = Root Mean Squared Error; MAE = Mean Absolute Error; ME = Mean Error. Bold values indicate the best-performing model for each metric and outcome. IERT: Intensive and Emerging Rehabilitation Therapies

Overall, the models exhibit stable and interpretable performance across a diverse set of health and economic outcomes. Their validity supports their application in individual-level economic evaluations and highlights the feasibility of integrating HRQoL and caregiver burden into broader value-based assessments of paediatric disability care.

Regarding the results of the nonparametric bootstrap resampling (1,000 iterations) further supported the robustness of the cost models. Bias-corrected and accelerated (BCa) confidence intervals showed high concordance with model-based estimates, validating the stability of the results and enhancing inference precision. More details on the coefficient estimates and BCa confidence intervals are available in the supplementary materials, see Tables S2-S6 for the Bootstrapping results of the cost models.

**Fig. 2:**
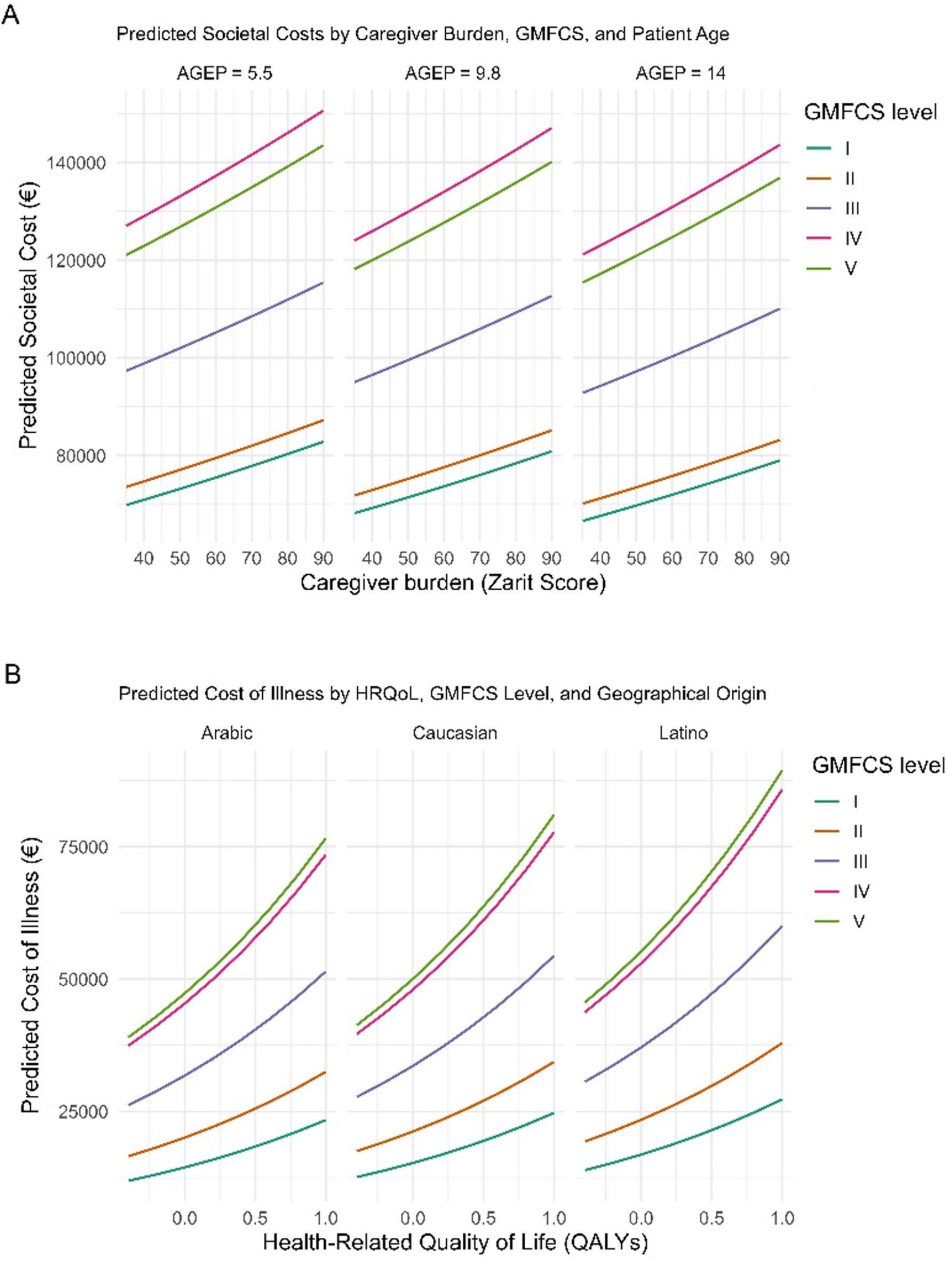

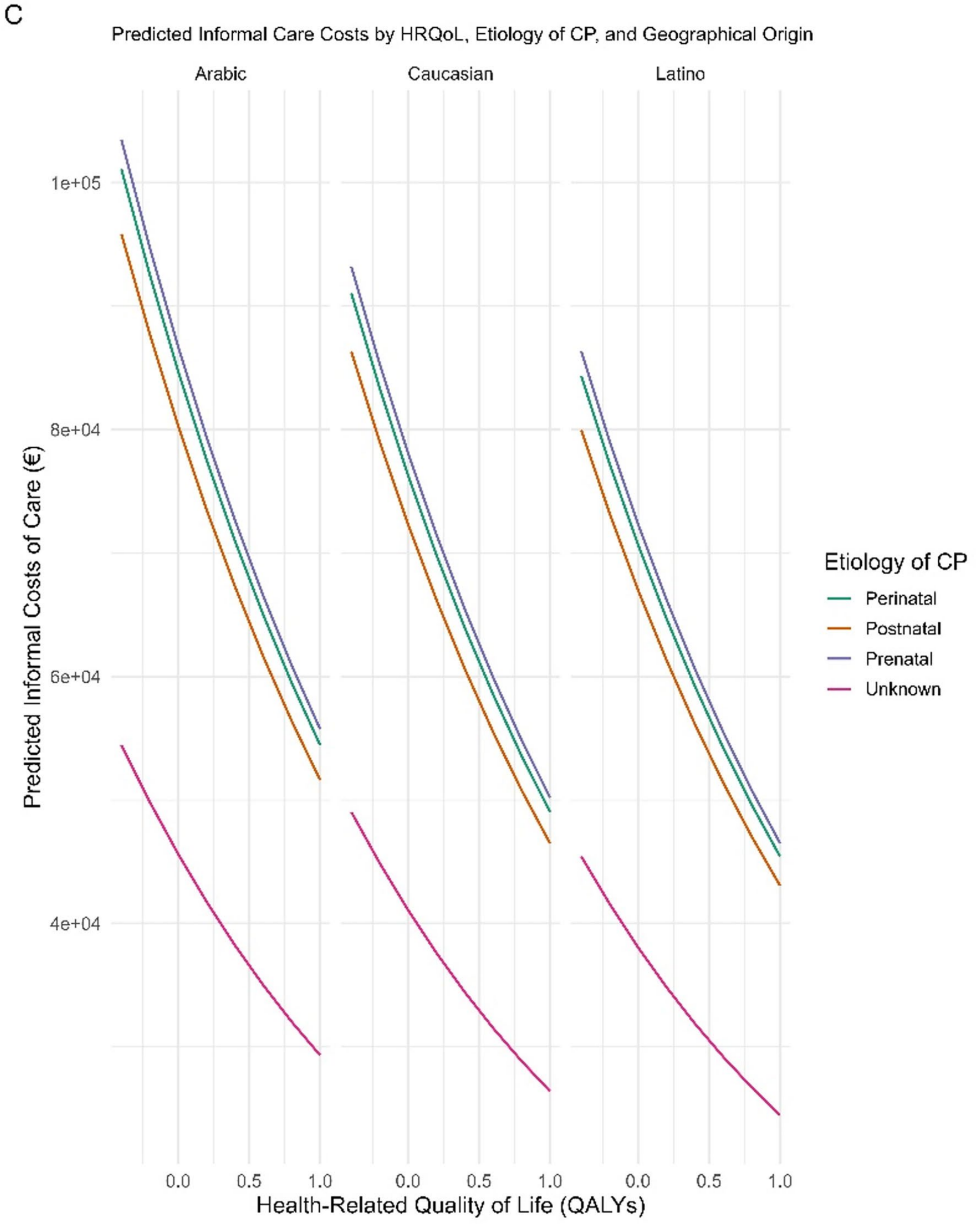
Predicted relationships between costs, health-related quality of life and key determinants for children with cerebral palsy, based on final models

Finally, as visualized in Fig. 2, the modelled associations between social costs, caregiver burden, functional severity, child age, HRQoL, and demographic factors exhibit complex and non-uniform patterns. Panel A shows a consistent increase in societal costs with higher caregiver burden (Zarit score), across all age groups. This relationship is modulated by motor severity (GMFCS level), with the highest costs observed in children classified as GMFCS III and IV, rather than the most severe level V. This counterintuitive pattern may reflect differential resource use or access barriers among families with the most functionally dependent children, possibly due to institutionalization or reduced access to formal services [28, 36].

Panels B and C reveal pronounced disparities in cost outcomes associated with health-related quality of life (HRQoL) and social determinants. In Panel B, lower HRQoL is strongly linked to higher illness-related costs, with Latino and Arab children incurring greater costs than their Spanish-born counterparts at equivalent impairment levels—suggesting inefficiencies rooted in structural or cultural barriers [12, 19, 23]. Panel C shows that informal care costs escalate as HRQoL declines, particularly among children with perinatal and postnatal etiologies. Notably, children with prenatal etiology appear to require less informal care, possibly due to earlier planning. Across both panels, minority backgrounds consistently align with steeper cost trajectories, underscoring the hidden burden borne by families with limited formal support [45]. These findings are not the result of ecological inference but arise from well-calibrated and internally validated predictive models that incorporate detailed clinical and contextual data.

### Development and application of a web-based calculator for individualized estimation

To facilitate the translation of model outputs into practice, we developed an interactive web-based calculator using R Shiny. This tool allows real-time estimation of annual societal costs and HRQoL outcomes based on specific clinical and sociodemographic profiles of children with CP and their caregivers. The calculator operates within the same 12-month analytic time horizon used in the empirical models. Health-related quality of life inputs corresponds to annual health utility values derived from the EQ-5D instruments, which are used to estimate QALYs accrued over a one-year period. Users may introduce decimal utility values (including negative values where applicable), consistent with the EQ-5D valuation framework.

The calculator is directly linked to the internally validated prediction models, ensuring consistency in the statistical structure, variable selection, and underlying assumptions. Users—including clinicians, social workers, and policy analysts—can input key variables (e.g., gross motor function level, caregiver burden, household income, type of schooling, access to services) and receive individualized projections of costs and HRQoL. This allows the simulation of alternative allocation scenarios, identification of high-burden profiles, and evaluation of budget impacts or cost-effectiveness at the person level, (See online Supplementary material).

To enhance usability and interpretability, the tool was pilot tested with clinicians and disability service coordinators prior to dissemination. Feedback from these stakeholders guided refinements in the interface and output formats, ensuring its relevance to real-world decision-making contexts.

Importantly, the calculator supports person-centred planning and equity-informed decision-making by making complex predictive analytics accessible and actionable. Users can simulate how changes in functional severity, social vulnerability, or service availability may influence projected outcomes, facilitating tailored resource allocation and prioritization. Further research is planned to assess the tool’s implementation in clinical and policy settings, including its potential to improve targeting of subsidies, optimize care pathways, and reduce disparities in paediatric disability management.

It is important to note that the calculator is based exclusively on internally validated models derived from the Navarra registry data. Although it enables individualized projections, its predictions should not be interpreted as causal effects nor generalized to other settings without external validation. The tool is intended to support exploratory analyses and hypothesis generation rather than to replace formal economic evaluations or policy decision frameworks.

## Discussion

This study provides robust, individual-level evidence on how clinical, sociodemographic, and socioeconomic factors jointly shape the distribution of costs and HRQoL among children with CP and their primary caregivers. Through internally validated predictive models, we show that functional severity, ethnic background, social class, and caregiver strain are key determinants of heterogeneous trajectories of economic burden and wellbeing [24]. This individualized approach contrasts with most aggregated cost-effectiveness analyses, which often obscure the complexity and inequity experienced by families affected by CP. From a health economics perspective, the observed associations between child health status and caregiver HRQoL may also be interpreted as *spillover effects*, whereby the health condition of one individual generates measurable impacts on the quality of life of family members or informal caregivers [8, 9]. Recognizing these spillover effects is increasingly considered important in economic evaluations, particularly in paediatric conditions where caregiving demands are substantial. This level of granularity is critical in paediatric disability, where the combination of functional impairment, social determinants, and caregiving dynamics generates non-linear and often inequitable patterns of burden [17, 23, 24]. These cross-cutting associations are visually summarized in a *predictor–outcome effect map* included in the Supplementary Material (Figure S1).

In our study, caregiver HRQoL was measured using EQ-5D-5L, complemented with the Zarit Burden Interview (ZBI), which captures emotional, physical, social, and financial dimensions of subjective caregiver burden. While this combination provides complementary insights, EQ-5D-5L alone may not fully reflect the multidimensional consequences of caregiving [46, 47]. Instruments such as the Caregiver Strain Index (CSI), Family Impact Scale (FIS), and Care-related Quality of Life instrument (CarerQoL) may provide more comprehensive assessments in future research. Notably, CarerQoL allows the derivation of utility values directly suitable for health technology assessment and economic evaluations, supporting the integration of caregiver outcomes into cost-utility analyses [6, 47].

Although our findings reflect the Spanish context, they are consistent with international evidence underscoring the substantial societal costs and economic burden of CP [36]. In Australia, Creelman et al. (2021) estimated the annual societal cost of CP as one of the highest among major childhood disabilities, highlighting the significant weight of out-of-pocket expenses, informal caregiving, and productivity losses. These cost components were strongly associated with functional severity (GMFCS levels), a pattern that closely mirrors our own results, where non-reimbursed family expenditures and unpaid care emerge as the principal cost drivers [7, 28].

In Canada, Amankwah et al. (2020) employed an individual-level microsimulation approach to model the long-term economic burden of CP, emphasizing the need for granular child-level data to capture the enduring complexity of care needs. Their findings reinforced that any refined economic modelling in this area must integrate both HRQoL and caregiver burden [17, 24]. Notably, their projections concluded that Canadians with CP will continue to experience reduced HRQoL, increased disability, and an escalating need for supportive services, particularly informal care.

Similarly, in the UK, Zhou and colleagues (2020–2024) have advanced cost modelling through individual-level approaches that demonstrate how functional severity and care context meaningfully reshape cost profiles [19, 23]. Their work underscores the critical value of disaggregated analyses. Our study contributes to this growing body of evidence by simultaneously incorporating child and caregiver HRQoL, offering a more comprehensive view of the societal burden of CP and enabling outcome estimation at the dyad level.

### Implications for policy and resource allocation

The study offers important implications for health policy and the equitable allocation of paediatric disability resources. First, the identification of out-of-pocket expenses, primarily for Intensive and Emerging Rehabilitation Therapies, as a major driver of cost heterogeneity reveals an informal privatization of care [14, 48]. These therapies, accessed by 64% of families in our sample, are concentrated among children with high motor impairment (GMFCS IV–V) and those living in areas with limited public service access [28, 49]. These findings suggest that policy discussions should consider the dual burden faced by families: the clinical need that motivates care-seeking and the financial strain it imposes on households. Failure to do so risks widening existing inequities by relegating potentially beneficial, albeit non-conventional therapies to those with greater financial means [6].

Second, the models show that total societal costs nearly double at higher GMFCS levels, and that socioeconomic and caregiving variables, including caregiver burden, household income, and schooling context substantially mediate cost and HRQoL outcomes [12, 28, 36]. These findings may inform the development of equity-sensitive reimbursement models, where eligibility for subsidies or service intensification is based not only on clinical severity, but also on social vulnerability and caregiving intensity [18, 50].

In this context, the Shiny calculator may serve as an exploratory decision-support tool, illustrating how predictive modelling could inform equity-sensitive allocation discussions within a 12-month analytic framework. By operationalizing individual-level data, the tool provides a structured framework that may support person-centred planning, while requiring external validation prior to broader policy application.

### Methodological utility of the predictive models

The predictive models developed in this study exhibit strong internal validity and practical relevance, supporting their potential use in research settings and exploratory policy analyses, pending external validation. Their performance across multiple domains reflects not only appropriate statistical specifications but also a rigorous variable selection process grounded in theory and empirical relevance. The models demonstrated excellent predictive accuracy in cross-validation, and their ability to capture complex interactions, such as the joint influence of motor severity, caregiver burden, and socioeconomic vulnerability, suggests their capacity to reflect clinically meaningful heterogeneity within the observed cohort [14, 17, 45]. Importantly, the models were designed with translational value in mind: they may be adapted for integration into microsimulation frameworks, distributional cost-effectiveness analyses, and equity-sensitive decision tools, subject to appropriate recalibration in new contexts [23].

In this context, the development of the interactive Shiny web calculator represents an illustrative translational output of this study, demonstrating how predictive modelling can be operationalized in a user-accessible format. However, its application should be considered exploratory, as model performance has not yet been assessed in external populations. These findings align with *family-centred outcomes* research by recognizing the interdependence between the health, economic, and psychosocial domains within caregiving dyads.

### External validity and generalisability

Although our sample represents the full paediatric CP population of Navarra, the transportability of these models to other settings requires careful consideration. Differences in regional financing structures, reimbursement policies, availability of rehabilitation services, and informal caregiving norms may alter cost compositions and the relationship between functional severity, socioeconomic vulnerability, and health utility outcomes.

Predictive performance may also be affected by distributional differences in key covariates, including GMFCS levels, access to non-reimbursed therapies, and baseline cost levels. Therefore, external validation in independent cohorts is necessary prior to application in other Spanish autonomous communities or international contexts.

Recalibration approaches, including intercept adjustment and model updating strategies, may improve performance in new settings. In line with structured validation frameworks such as AdViSHE [51], future work should assess model validity across conceptual, technical, and external domains before broader implementation. The availability of the R code facilitates transparent replication and recalibration in other jurisdictions.

### Study limitations

This study has some important limitations. First, the cross-sectional design precludes causal inference; although extensive covariate adjustment was applied, the possibility of unmeasured confounding or reverse causality cannot be ruled out. Second, child HRQoL was proxy-reported by caregivers, a common practice in paediatric disability research, particularly when communication or cognitive impairments prevent self-reporting [12, 13]. However, this approach may introduce systematic bias, as caregiver perceptions can be influenced by emotional distress, expectations, or burden. Prior studies have shown only moderate agreement between self- and proxy-reported HRQoL, especially in children with severe impairments [52].

In addition, while caregiver HRQoL was measured using EQ-5D-5L and subjective burden assessed with the ZBI, this combination provides complementary insights but does not fully capture the multidimensional impacts of caregiving, including emotional, physical, and social domains [46, 47]. Future studies could incorporate instruments specifically designed for caregiver outcomes, such as the CSI, FIS, or CarerQoL, to allow a more comprehensive representation of the burden of caring for children with CP and to inform interventions and policy measures that are more family centred.

Third, the component labelled Intensive and Emerging Rehabilitation Therapies comprises a heterogeneous set of interventions (e.g., neurorehabilitation, aquatic therapy, robotic-assisted therapy) that vary in standardization and evidence base [48], limiting the interpretability of results for specific therapies [17, 53]. Fourth, while the sample was exhaustive within a single Spanish region (Navarra), generalizability to other regions or countries may be limited due to variation in public benefits, health infrastructure, and sociocultural norms surrounding disability and care [6, 7, 50].

Finally, although the predictive models demonstrated strong internal validity through repeated cross-validation, external validation in independent populations remains necessary to assess generalizability and support broader implementation. Importantly, the Shiny web calculator developed in this study should be regarded as an exploratory decision-support prototype rather than a fully validated policy tool. Its estimates of individualized costs and HRQoL are based solely on the Navarra dataset, and their application to other contexts may be limited. Users are advised to interpret the predictions with caution, and further validation with external data is required before using the tool for policy or clinical decision-making.

### Strengths and contributions

Despite these limitations, the study provides a methodologically rigorous and policy-relevant framework for modelling costs and outcomes in paediatric CP: (1) It is the first study in Spain to jointly model disaggregated costs and HRQoL at the individual level using real-world data from a population-based registry. (2) It integrates seven clinical functional classification systems, allowing for highly specific modelling of child heterogeneity. (3) It incorporates caregiver HRQoL and burden, recognizing the dyadic nature of paediatric disability. (4) It addresses structural and territorial inequities, offering insights into how geography and ethnicity shape health outcomes and costs. (5) It offers a public, user-friendly tool for cost estimation and policy simulation, designed to support exploratory analyses and future methodological development rather than immediate policy implementation.

In sum, this study applies a bidirectional modelling strategy to examine how costs and quality of life influence each other within the caregiving dyad. By integrating real-world data on HRQoL, caregiver burden, and disaggregated cost categories, it captures the interdependencies that shape well-being in families affected by paediatric cerebral palsy. The models show that child QALYs significantly predict informal care needs and out-of-pocket expenditures, while investment in non-standard therapies is associated with improved child HRQoL. Conversely, high financial strain contributes to increased caregiver burden and reduced caregiver QALYs, illustrating a circular dynamic of vulnerability.

These findings provide an analytical foundation for future equity-sensitive evaluations that prioritize person-centred care and explicitly consider health utility as a key outcome in disability management. The publicly accessible decision-support tool demonstrates how such modelling approaches may inform structured allocation discussions, contingent upon appropriate external validation and contextual adaptation.

## Conclusions

This study provides new empirical evidence on the economic and quality-of-life impact of paediatric CP in Spain, integrating individual-level cost modelling and health utility prediction for both children and their caregivers. By identifying the out-of-pocket burden associated with Intensive and Emerging Rehabilitation Therapies and quantifying its association with HRQoL, this research highlights the need to expand the scope of economic evaluations beyond publicly funded services. The models developed capture real-world heterogeneity and offer a robust framework for supporting person-centred decision-making and resource allocation in paediatric disability care.

Nevertheless, the predictive models developed in this study should be interpreted as internally validated tools derived from a single regional population. While they offer valuable insights into individual-level heterogeneity in costs and health utility, their application beyond the Navarra context requires external validation and recalibration. The accompanying Shiny calculator should therefore be considered a decision-support prototype designed to illustrate how predictive modelling can inform person-centred planning. Its outputs represent model-based annual projections rather than definitive cost or long-term QALY estimates. Future research should extend this framework through longitudinal validation, cross-regional adaptation, and model-based simulations to assess the long-term cost-effectiveness of alternative care strategies. Strengthening the external validity and scalability of these tools will be essential to support more equitable, efficient, and evidence-informed paediatric disability systems in diverse settings.

## Supplementary Information

Below is the link to the electronic supplementary material.

Supplementary Material 1

Supplementary Material 2

## Data Availability

The full dataset of this study is available upon reasonable and justified request to the corresponding author and the data-holding institution (Hospital Universitario de Navarra). However, most of the data required to replicate the results are publicly available in the GitHub repository.
