# Supplementary material for: Individual-level prediction models of societal costs and health-related quality of life in pediatric cerebral palsy: a population-based study from Spain
Source: Cost Eff Resour Alloc. 2026 Apr 11;24:72. doi: 10.1186/s12962-026-00743-y (PMC13224597; doi:10.1186/s12962-026-00743-y)
Supplement: Supplementary file 1 — Supplementary Material 1 [file 12962_2026_743_MOESM1_ESM.docx]

**Supplementary Material**

**Table S1.** Baseline characteristics of study participants

| ***Analysis of out-of-pocket healthcare costs Mean (SD) or n (%) **** | | | |
| --- | --- | --- | --- |
| **Characteristics** | **Overall N= 148** | **With Out-of-pocket Healthcare Cost *N=92*** | **Without Out-of-pocket**  **Healthcare cost  N=56** |
| Age of caregiver | 42.97 (6.25) | 43.04 (6.29) | 42.86 (6.25) |
| Age of child | 9.78 (4.25) | 10.05 (4.31) | 9.32 (4.14) |
| *Gender of caregiver* |  |  |  |
| Female | 98 (66%) | 58 (63%) | 40 (71%) |
| Male | 50 (34%) | 34 (37%) | 16 (29%) |
| *Gender of child* |  |  |  |
| Female | 70 (47%) | 46 (50%) | 24 (43%) |
| Male | 78 (53%) | 46 (50%) | 32 (57%) |
| *Marital status of caregiver* |  |  |  |
| Divorced | 18 (12%) | 11 (12%) | 7 (13%) |
| Married | 106 (72%) | 71 (77%) | 35 (63%) |
| Single | 24 (16%) | 10 (11%) | 14 (25%) |
| *Caregiver's profession* |  |  |  |
| Accounting, administrative and other office workers | 21 (14%) | 16 (17%) | 5 (8.9%) |
| Agricultural workers | 2 (1.4%) | 1 (1.1%) | 1 (1.8%) |
| Catering and retail workers | 30 (20%) | 16 (17%) | 14 (25%) |
| Directors and managers | 7 (4.7%) | 3 (3.3%) | 4 (7.1%) |
| Homemaker and domestic service | 24 (16%) | 13 (14%) | 11 (20%) |
| Plant and machinery operators and assemblers | 18 (12%) | 12 (13%) | 6 (11%) |
| Skilled workers in the manufacturing and construction industries | 6 (4.1%) | 5 (5.4%) | 1 (1.8%) |
| Technical and professional, scientific or intellectual | 40 (27%) | 26 (28%) | 14 (25%) |
| *Education level of the caregiver* |  |  |  |
| Elementary | 15 (10%) | 6 (6.5%) | 9 (16%) |
| Secondary | 25 (17%) | 17 (18%) | 8 (14%) |
| Tertiary | 108 (73%) | 69 (75%) | 39 (70%) |
| *Geographical origin of the caregiver* |  |  |  |
| Arabic | 23 (16%) | 7 (7.6%) | 16 (29%) |
| Caucasian | 107 (72%) | 71 (77%) | 36 (64%) |
| Latin American | 18 (12%) | 14 (15%) | 4 (7.1%) |
| *Gave up/loosed job because of child's disability* | 73 (49%) | 51 (55%) | 22 (39%) |
| *Annual household income* | 40,409.73 (17,785.52) | 41,419.13 (16,675.38) | 38,751.43 (19,515.87) |
| *Social Class* |  |  |  |
| Hight | 16 (11%) | 8 (8.7%) | 8 (14%) |
| Low | 23 (16%) | 11 (12%) | 12 (21%) |
| Lower Middle | 41 (28%) | 25 (27%) | 16 (29%) |
| Middle | 68 (46%) | 48 (52%) | 20 (36%) |
| *Type of school the child attends* |  |  |  |
| Normal | 36 (24%) | 18 (20%) | 18 (32%) |
| Normal with support | 35 (24%) | 20 (22%) | 15 (27%) |
| Special center | 55 (37%) | 42 (46%) | 13 (23%) |
| Special class | 22 (15%) | 12 (13%) | 10 (18%) |
| *Child's level of education* |  |  |  |
| Elementary | 45 (30%) | 23 (25%) | 22 (39%) |
| Kindergarden | 20 (14%) | 14 (15%) | 6 (11%) |
| Prof education | 3 (2.0%) | 3 (3.3%) | 0 (0%) |
| Secondary | 27 (18%) | 16 (17%) | 11 (20%) |
| Special Education | 53 (36%) | 36 (39%) | 17 (30%) |
| **Table S1.** Baseline characteristics of study participants (Continued) |  |  |  |
| ***Analysis of out-of-pocket healthcare costs Mean (SD) or n (%) **** |  |  |  |
| **Characteristics** | **Overall N= 148** | **With Out-of-pocket Healthcare Cost *N=92*** | **Without Out-of-pocket**  **Healthcare cost  N=56** |
| *Functional classification systems* |  |  |  |
| GMFCS (Gross Motor Function Classification System) |  |  |  |
| I | 24 (16%) | 7 (7.6%) | 17 (30%) |
| II | 35 (24%) | 16 (17%) | 19 (34%) |
| III | 29 (20%) | 20 (22%) | 9 (16%) |
| IV | 21 (14%) | 18 (20%) | 3 (5.4%) |
| V | 39 (26%) | 31 (34%) | 8 (14%) |
| VSS (Viking Speech Scale) |  |  |  |
| I | 50 (34%) | 30 (33%) | 20 (36%) |
| II | 26 (18%) | 12 (13%) | 14 (25%) |
| III | 18 (12%) | 10 (11%) | 8 (14%) |
| IV | 54 (36%) | 40 (43%) | 14 (25%) |
| VFCS (Visual Function Classification System) |  |  |  |
| I | 56 (38%) | 31 (34%) | 25 (45%) |
| II | 35 (24%) | 20 (22%) | 15 (27%) |
| III | 17 (11%) | 13 (14%) | 4 (7.1%) |
| IV | 15 (10%) | 7 (7.6%) | 8 (14%) |
| V | 25 (17%) | 21 (23%) | 4 (7.1%) |
| MACS (Manual Ability Classification System) |  |  |  |
| I | 33 (22%) | 16 (17%) | 17 (30%) |
| II | 44 (30%) | 23 (25%) | 21 (38%) |
| III | 18 (12%) | 12 (13%) | 6 (11%) |
| IV | 22 (15%) | 17 (18%) | 5 (8.9%) |
| V | 31 (21%) | 24 (26%) | 7 (13%) |
| EDACS (Eating and Drinking Ability Classification System) |  |  |  |
| I | 56 (38%) | 33 (36%) | 23 (41%) |
| II | 27 (18%) | 13 (14%) | 14 (25%) |
| III | 22 (15%) | 14 (15%) | 8 (14%) |
| IV | 18 (12%) | 12 (13%) | 6 (11%) |
| V | 25 (17%) | 20 (22%) | 5 (8.9%) |
| CFCS (Communication Function Classification System) |  |  |  |
| I | 51 (34%) | 29 (32%) | 22 (39%) |
| II | 23 (16%) | 10 (11%) | 13 (23%) |
| III | 24 (16%) | 17 (18%) | 7 (13%) |
| IV | 23 (16%) | 16 (17%) | 7 (13%) |
| V | 27 (18%) | 20 (22%) | 7 (13%) |
| BFMF (Bimanual Fine Motor Function) |  |  |  |
| I | 34 (23%) | 15 (16%) | 19 (34%) |
| IIa | 20 (14%) | 13 (14%) | 7 (13%) |
| IIb | 22 (15%) | 12 (13%) | 10 (18%) |
| IIIa | 7 (4.7%) | 5 (5.4%) | 2 (3.6%) |
| IIIb | 13 (8.8%) | 6 (6.5%) | 7 (13%) |
| Iva | 9 (6.1%) | 7 (7.6%) | 2 (3.6%) |
| IVb | 12 (8.1%) | 11 (12%) | 1 (1.8%) |
| V | 31 (21%) | 23 (25%) | 8 (14%) |
| *Type of Cerebral Palsy* |  |  |  |
| Ataxic | 8 (5.4%) | 5 (5.4%) | 3 (5.4%) |
| Dyskinetic | 8 (5.4%) | 6 (6.5%) | 2 (3.6%) |
| Mixed | 15 (10%) | 14 (15%) | 1 (1.8%) |
| Spastic | 117 (79%) | 67 (73%) | 50 (89%) |
| **Table S1.** Baseline characteristics of study participants (Continued) |  |  |  |
| ***Analysis of out-of-pocket healthcare costs Mean (SD) or n (%) **** |  |  |  |
| **Characteristics** | **Overall N= 148** | **With Out-of-pocket Healthcare Cost *N=92*** | **Without Out-of-pocket**  **Healthcare cost  N=56** |
| *Etiology or origin of cerebral palsy* |  |  |  |
| Perinatal | 72 (49%) | 43 (47%) | 29 (52%) |
| Postnatal | 16 (11%) | 12 (13%) | 4 (7.1%) |
| Prenatal | 55 (37%) | 35 (38%) | 20 (36%) |
| Unknown | 5 (3.4%) | 2 (2.2%) | 3 (5.4%) |
| *Impairment index* |  |  |  |
| High | 61 (41%) | 45 (49%) | 16 (29%) |
| Low | 43 (29%) | 22 (24%) | 21 (38%) |
| Moderate | 44 (30%) | 25 (27%) | 19 (34%) |
| *MRICS (Magnetic Resonance Imaging Classification System)* |  |  |  |
| A | 27 (18%) | 19 (21%) | 8 (14%) |
| B | 34 (23%) | 22 (24%) | 12 (21%) |
| C | 55 (37%) | 32 (35%) | 23 (41%) |
| D | 19 (13%) | 8 (8.7%) | 11 (20%) |
| E | 13 (8.8%) | 11 (12%) | 2 (3.6%) |
| *Place of residence* |  |  |  |
| Easy access to health services | 80 (54%) | 55 (60%) | 25 (45%) |
| Limited access to health services | 40 (27%) | 26 (28%) | 14 (25%) |
| No local access to health services | 28 (19%) | 11 (12%) | 17 (30%) |
| ** The out-of-pocket health care cost in children with CP refers to what the patient spends on complementary and alternative medicine, outside the health care system, resulting in excess cost of illness.* | | | |

**Bootstrapping Results Cost Models**

**Table S2. Model 1 Cost of informal care, bootstrap results**

**IC Bootstrap 95% - model_Ycare**

| Variable | Estimate | Perc_low | Perc_high | BCa_low | BCa_high | Signif |
| --- | --- | --- | --- | --- | --- | --- |
| (Intercept) | 10.6389 | 10.2991 | 10.9804 | 10.2990 | 10.9804 | * |
| SEXCM | -0.0859 | -0.1842 | 0.0225 | -0.1881 | 0.0191 |  |
| GOICaucasian | -0.1049 | -0.2406 | 0.0259 | -0.2220 | 0.0498 |  |
| GOILatino | -0.1813 | -0.3556 | -0.0324 | -0.3381 | -0.0169 | * |
| CFCSII | -0.1420 | -0.2869 | 0.0103 | -0.2962 | -0.0013 | * |
| CFCSIII | -0.0318 | -0.1922 | 0.1197 | -0.1935 | 0.1181 |  |
| CFCSIV | 0.1140 | -0.0366 | 0.2536 | -0.0485 | 0.2440 |  |
| CFCSV | -0.0367 | -0.1806 | 0.1304 | -0.1953 | 0.1121 |  |
| ETIOLPostnatal | -0.0534 | -0.1689 | 0.0565 | -0.1693 | 0.0556 |  |
| ETIOLPrenatal | 0.0234 | -0.1248 | 0.1568 | -0.1163 | 0.1628 |  |
| ETIOLUnknown | -0.6190 | -1.0514 | -0.1846 | -1.0103 | -0.1136 | * |
| MRICSB | -0.0591 | -0.2394 | 0.1238 | -0.2503 | 0.1120 |  |
| MRICSC | -0.0420 | -0.1925 | 0.0975 | -0.1918 | 0.0980 |  |
| MRICSD | 0.2139 | 0.0736 | 0.3650 | 0.0485 | 0.3484 | * |
| MRICSE | 0.2626 | 0.1182 | 0.4116 | 0.1326 | 0.4237 | * |
| Zarith_Score | 0.0078 | 0.0039 | 0.0122 | 0.0038 | 0.0120 | * |
| z_QALYsC | -0.1312 | -0.2000 | -0.0600 | -0.2036 | -0.0645 | * |

CI = 95% Confidence Interval. BCa: bias-corrected and accelerated bootstrap confidence intervals. Asterisks indicate coefficients with BCa intervals that do not include zero, interpreted as statistically significant at the 95% level.

(Intercept): model intercept. SEXCM: male caregiver. GOICaucasian: Caucasian geographic origin. GOILatino: Latin American geographic origin. CFCSII–V: levels II to V of the Communication Function Classification System. ETIOLPostnatal/Prenatal/Unknown: postnatal, prenatal, and unknown etiology, respectively. MRICSB–E: levels B to E of the Magnetic Resonance Imaging Classification System. *Zarith_Score,* represent the Zarit Burden Interview total score of caregivers. z_QALYsC: standardized QALYs gained by children with cerebral palsy (CP).

**Table S3. Model 2 Social Cost bootstrap results**

**IC Bootstrap 95% - model_SocialCost**

| Variable | Estimate | Perc_low | Perc_high | BCa_low | BCa_high | Signif |
| --- | --- | --- | --- | --- | --- | --- |
| (Intercept) | 11.0220 | 10.8465 | 11.2271 | 10.8313 | 11.2014 | * |
| SEXPM | -0.0352 | -0.0958 | 0.0137 | -0.0821 | 0.0215 |  |
| SEXCM | -0.0411 | -0.1080 | 0.0170 | -0.1053 | 0.0218 |  |
| GMFCSII | 0.0518 | -0.0405 | 0.1709 | -0.0427 | 0.1655 |  |
| GMFCSIII | 0.3319 | 0.2239 | 0.4524 | 0.2190 | 0.4485 | * |
| GMFCSIV | 0.5982 | 0.4518 | 0.7520 | 0.4520 | 0.7523 | * |
| GMFCSV | 0.5499 | 0.4028 | 0.6997 | 0.3928 | 0.6963 | * |
| ETIOLPostnatal | 0.0094 | -0.0709 | 0.0932 | -0.0705 | 0.0934 |  |
| ETIOLPrenatal | -0.0032 | -0.0913 | 0.0813 | -0.0975 | 0.0768 |  |
| ETIOLUnknown | -0.3427 | -0.5822 | -0.1013 | -0.5755 | -0.0914 | * |
| MRICSB | -0.0314 | -0.1374 | 0.0862 | -0.1394 | 0.0800 |  |
| MRICSC | 0.0058 | -0.0796 | 0.0924 | -0.0832 | 0.0837 |  |
| MRICSD | 0.0721 | -0.0117 | 0.1663 | -0.0245 | 0.1508 |  |
| MRICSE | 0.1818 | 0.0645 | 0.2911 | 0.0700 | 0.3011 | * |
| Zarith_Score | 0.0031 | -0.0005 | 0.0066 | -0.0003 | 0.0069 |  |
| z_AGEP | -0.0238 | -0.0508 | 0.0037 | -0.0509 | 0.0036 |  |

CI = 95% Confidence Interval. BCa: bias-corrected and accelerated bootstrap confidence intervals. Asterisks indicate coefficients with BCa intervals that do not include zero, interpreted as statistically significant at the 95% level.

The variables are defined as follows: (Intercept) represents the model intercept; SEXPM and SEXCM indicate male child and male caregiver, respectively; GMFCSII to GMFCSV correspond to levels II through V of the Gross Motor Function Classification System; ETIOLPostnatal, ETIOLPrenatal, and ETIOLUnknown denote postnatal, prenatal, and unknown etiology, respectively; MRICSB to MRICSE refer to levels B through E of the Magnetic Resonance Imaging Classification System. *Zarith_Score,* represent the Zarit Burden Interview total score of caregivers. Lastly, z_AGEP is the child's age standardized using a z-score transformation.

**Table S4. Model 3 Cost of Illness bootstrap results**

**IC Bootstrap 95% - model_CostsCP**

| Variable | Estimate | Perc_low | Perc_high | BCa_low | BCa_high | Signif |
| --- | --- | --- | --- | --- | --- | --- |
| (Intercept) | 9.8418 | 9.6967 | 9.9827 | 9.7043 | 9.9862 | * |
| SEXPM | -0.0620 | -0.1312 | -0.0018 | -0.1286 | -0.0004 | * |
| GMFCSII | 0.3286 | 0.2110 | 0.4251 | 0.2391 | 0.4510 | * |
| GMFCSIII | 0.7881 | 0.6675 | 0.9068 | 0.6702 | 0.9080 | * |
| GMFCSIV | 1.1446 | 0.9442 | 1.3352 | 0.9321 | 1.3212 | * |
| GMFCSV | 1.1859 | 0.9338 | 1.4307 | 0.9019 | 1.4008 | * |
| GOICaucasian | 0.0563 | -0.0583 | 0.1548 | -0.0470 | 0.1639 |  |
| GOILatino | 0.1551 | 0.0402 | 0.2757 | 0.0316 | 0.2696 | * |
| SCH_SUPPNormal with support | -0.0147 | -0.1039 | 0.0742 | -0.1035 | 0.0750 |  |
| SCH_SUPPSpecial center | -0.0865 | -0.2202 | 0.0529 | -0.2208 | 0.0527 |  |
| SCH_SUPPSpecial class | 0.0390 | -0.0977 | 0.1603 | -0.0991 | 0.1554 |  |
| VSSII | -0.1868 | -0.3310 | -0.0115 | -0.3339 | -0.0170 | * |
| VSSIII | -0.1508 | -0.3244 | 0.0370 | -0.3284 | 0.0311 |  |
| VSSIV | -0.1106 | -0.3023 | 0.1067 | -0.3189 | 0.0916 |  |
| VFCSII | 0.0306 | -0.1015 | 0.1611 | -0.1020 | 0.1593 |  |
| VFCSIII | 0.1091 | -0.0504 | 0.2320 | -0.0568 | 0.2285 |  |
| VFCSIV | -0.0915 | -0.2519 | 0.0670 | -0.2641 | 0.0555 |  |
| VFCSV | -0.0848 | -0.2800 | 0.0965 | -0.2775 | 0.0978 |  |
| EDACSII | -0.0016 | -0.1601 | 0.1800 | -0.1538 | 0.1963 |  |
| EDACSIII | -0.1025 | -0.2575 | 0.0553 | -0.2376 | 0.0872 |  |
| EDACSIV | -0.0771 | -0.3129 | 0.1239 | -0.3019 | 0.1287 |  |
| EDACSV | 0.1010 | -0.1911 | 0.3523 | -0.1638 | 0.3698 |  |
| CFCSII | 0.1908 | 0.0539 | 0.3297 | 0.0551 | 0.3302 | * |
| CFCSIII | 0.2343 | 0.0572 | 0.3896 | 0.0503 | 0.3816 | * |
| CFCSIV | 0.2384 | 0.0086 | 0.4645 | 0.0112 | 0.4734 | * |
| CFCSV | 0.1971 | -0.0512 | 0.4533 | -0.0473 | 0.4568 |  |
| ETIOLPostnatal | 0.0724 | -0.0382 | 0.1985 | -0.0493 | 0.1841 |  |
| ETIOLPrenatal | -0.0854 | -0.1538 | -0.0100 | -0.1544 | -0.0108 | * |
| ETIOLUnknown | 0.0838 | -0.0927 | 0.2966 | -0.0921 | 0.3028 |  |
| z_AGEC | -0.0331 | -0.0724 | 0.0022 | -0.0693 | 0.0038 |  |
| z_HOUSEINCY | 0.0381 | -0.0042 | 0.0817 | -0.0135 | 0.0767 |  |
| z_QALYsA | -0.0365 | -0.0938 | 0.0171 | -0.0967 | 0.0124 |  |
| z_QALYsC | 0.1429 | 0.0857 | 0.2069 | 0.0778 | 0.1991 | * |

CI = 95% Confidence Interval. BCa: bias-corrected and accelerated bootstrap confidence intervals. Asterisks indicate coefficients with BCa intervals that do not include zero, interpreted as statistically significant at the 95% level.

The variables are defined as follows: (Intercept) represents the model intercept; SEXPM indicates a male child. GMFCSII to GMFCSV refer to levels II through V of the Gross Motor Function Classification System. GOICaucasian and GOILatino denote the child’s Caucasian or Latino geographical origin, respectively. SCH_SUPPNormal with support, SCH_SUPPSpecial center, and SCH_SUPPSpecial class indicate school support settings: mainstream school with support, special education center, and special classroom within a mainstream school, respectively. VSSII to VSSIV correspond to levels II through IV of the Viking Speech Scale. VFCSII to VFCSV refer to levels II through V of the Visual Function Classification Scale. EDACSII to EDACSV represent levels II through V of the Eating and Drinking Ability Classification System. CFCSII to CFCSV indicate levels II through V of the Communication Function Classification System. ETIOLPostnatal, ETIOLPrenatal, and ETIOLUnknown denote postnatal, prenatal, and unknown etiology of cerebral palsy, respectively. z_AGEC is the caregiver´s age standardized using a z-score transformation; z_HOUSEINCY is the standardized annual household income; z_QALYsA and z_QALYsC refer to the standardized Quality-Adjusted Life Years (QALYs) reported for the caregiver and the child, respectively.

**Table S5. Model 4 Government Costs bootstrap results**

| **IC Bootstrap 95% - Model Government Costs** | | | | | |  |
| --- | --- | --- | --- | --- | --- | --- |
| Variable | Estimate | Perc_low | Perc_high | BCa_low | BCa_high | Signif |
| (Intercept) | 8.8994 | 8.6456 | 9.1414 | 8.6459 | 9.1415 | * |
| GMFCSII | 0.3074 | 0.1396 | 0.4870 | 0.1279 | 0.4829 | * |
| GMFCSIII | 0.7268 | 0.5869 | 0.8887 | 0.5846 | 0.8870 | * |
| GMFCSIV | 0.9343 | 0.7435 | 1.1320 | 0.7400 | 1.1307 | * |
| GMFCSV | 1.0556 | 0.8577 | 1.2636 | 0.8534 | 1.2616 | * |
| CIVISTATMarried | -0.0201 | -0.1057 | 0.0893 | -0.1030 | 1.1003 |  |
| CIVISTATSingle | 0.1211 | -0.0244 | 0.2692 | -0.0176 | 0.2759 |  |
| Zarith_Score | -0.0015 | -0.0061 | 0.0035 | -0.0058 | 0.0038 |  |
| CI = 95% Confidence Interval. BCa: bias-corrected and accelerated bootstrap confidence intervals. Asterisks indicate coefficients with BCa intervals that do not include zero, interpreted as statistically significant at the 95% level. The variables are defined as follows: (Intercept) represents the model intercept. GMFCSII to GMFCSV refer to levels II through V of the Gross Motor Function Classification System. CIVISTATMarried and CIVISTATSingle indicate the caregiver's civil status as married or single, respectively. *Zarith_Score,* represent the Zarit Burden Interview total score of caregivers, which measures caregiver burden. | | | | | | |
|  |  |  |  |  |  |  |

**Table S6. Model 5 Medical Care Cost bootstrap results**

**IC Bootstrap 95% - model_MedCare**

| Variable | Estimate | Perc_low | Perc_high | BCa_low | BCa_high | Signif |
| --- | --- | --- | --- | --- | --- | --- |
| (Intercept) | 8.3390 | 8.2410 | 8.4463 | 8.2453 | 8.4546 | * |
| SEXCM | -0.0873 | -0.1729 | -0.0094 | -0.1639 | -0.0022 | * |
| SEXPM | -0.0971 | -0.1860 | -0.0043 | -0.1869 | -0.0059 | * |
| EDACSII | -0.0003 | -0.1185 | 0.1288 | -0.1061 | 0.1388 |  |
| EDACSIII | -0.1568 | -0.2637 | -0.0450 | -0.2724 | -0.0492 | * |
| EDACSIV | -0.0690 | -0.2171 | 0.0711 | -0.2210 | 0.0685 |  |
| EDACSV | 0.0360 | -0.1577 | 0.2290 | -0.1548 | 0.2381 |  |
| z_QALYsA | -0.0945 | -0.1534 | -0.0355 | -0.1563 | -0.0395 | * |

CI = 95% Confidence Interval. BCa: bias-corrected and accelerated bootstrap confidence intervals. Asterisks indicate coefficients with BCa intervals that do not include zero, interpreted as statistically significant at the 95% level.

The variables are defined as follows: (Intercept) represents the model intercept. SEXCM and SEXPM indicate male sex of the caregiver and the child, respectively. EDACSII to EDACSV refer to levels II through V of the Eating and Drinking Ability Classification System. z_QALYsA represents standardized QALYs gained by the caregiver.

**
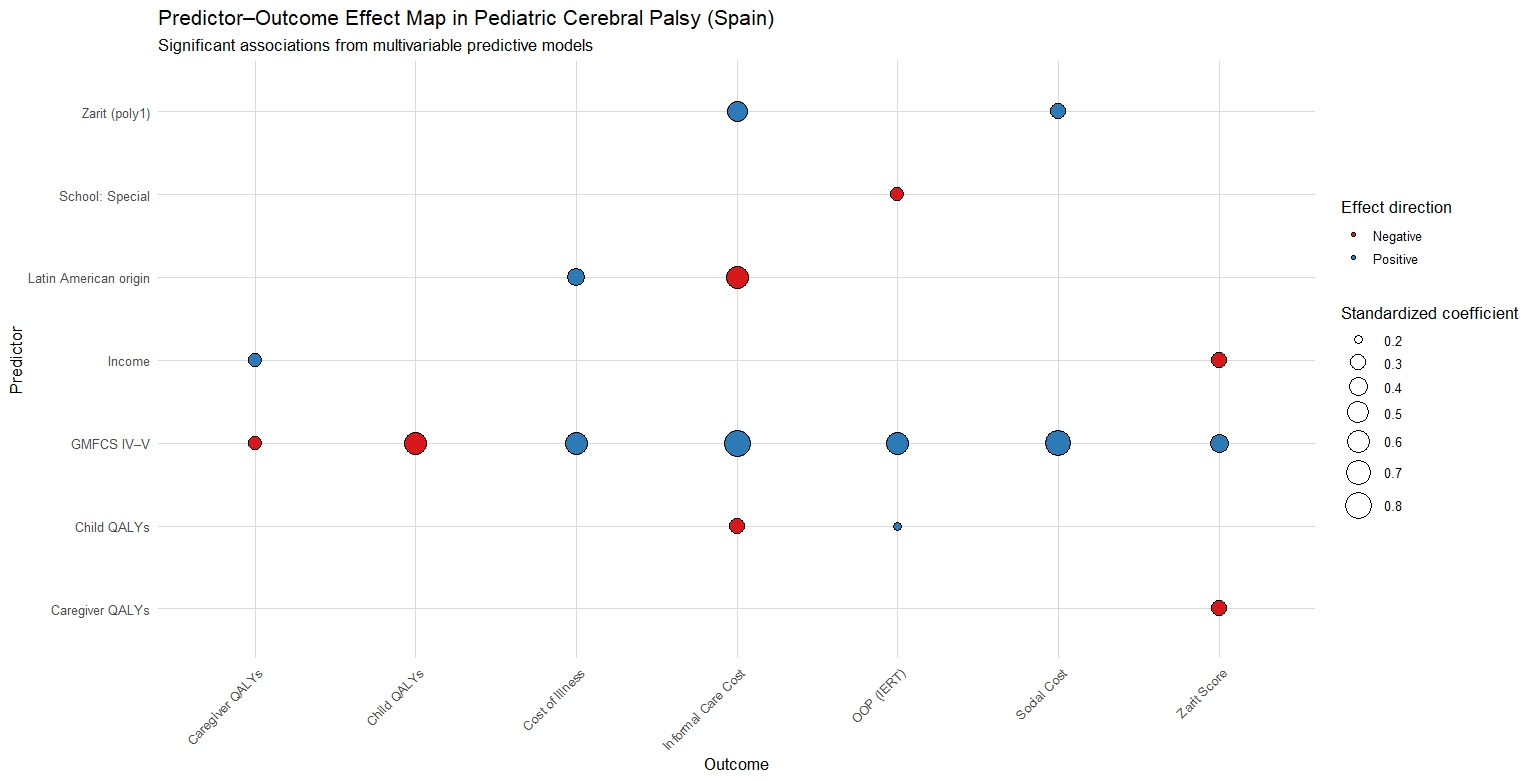
****Figure S1**. Predictor–Outcome Effect Map from Individual-Level Models in Pediatric Cerebral Palsy (Spain)

**Interpretation:** The GMFCS levels IV–V were consistently associated with higher costs (informal, OOP, total) and lower HRQoL for both children and caregivers, indicating a bidirectional burden. Caregiver burden (Zarit score) was positively associated with informal and social costs and negatively with caregiver HRQoL. Latin American origin was linked to greater economic burden. Household income and schooling context shaped both quality of life and financial outcomes. This figure summarizes the complex interplay between child impairment, caregiving strain, and structural factors in shaping health and economic trajectories.

**Online Supplementary Material**

The online version includes additional materials containing the R scripts used to generate the results of the article, structured in six steps. It also provides access to an interactive Shiny calculator developed to validate the findings in external populations. This tool is intended to support decision-makers in health policy, researchers, and clinical professionals. Available at:

- GitHub repository: <https://github.com/Diana-MND1996/Modelling-the-Interplay-Between-Quality-of-Life-and-Societal-Costs>

The web-based calculator includes an input field labelled **“Annual health utility (EQ-5D value)”**, representing the preference-weighted HRQoL value used to estimate QALYs accrued over a one-year period within the model.

- Shiny Calculator: <https://diananovacostshrqol.shinyapps.io/costs_cp/>
