# Supplementary material for: Individual-level prediction models of societal costs and health-related quality of life in pediatric cerebral palsy: a population-based study from Spain
Source: Cost Eff Resour Alloc. 2026 Apr 11;24:72. doi: 10.1186/s12962-026-00743-y (PMC13224597; doi:10.1186/s12962-026-00743-y)
Supplement: Supplementary file 2 — Supplementary Material 2 [file 12962_2026_743_MOESM2_ESM.docx]

**Supplementary Material**

The online version includes additional materials containing the R scripts used to generate the results of the article, structured in six steps. It also provides access to an interactive Shiny calculator developed to validate the findings in external populations. This tool is intended to support decision-makers in health policy, researchers, and clinical professionals. Available at:

- GitHub repository: <https://github.com/Diana-MND1996/Modelling-the-Interplay-Between-Quality-of-Life-and-Societal-Costs>
- Calculator: <https://diananovacostshrqol.shinyapps.io/costs_cp/>
